# Mega-scale Bayesian regression methods for genome-wide prediction and association studies with thousands of traits

**DOI:** 10.1093/genetics/iyac183

**Published:** 2022-12-19

**Authors:** Jiayi Qu, Daniel Runcie, Hao Cheng

**Affiliations:** Department of Animal Science, University of California Davis, Davis, CA 95616, USA; Department of Plant Sciences, University of California Davis, Davis, CA 95616, USA; Department of Plant Sciences, University of California Davis, Davis, CA 95616, USA

**Keywords:** multi-trait, genomic prediction, genome-wide association studies, high-throughput phenotyping, Bayesian regression models

## Abstract

Large-scale phenotype data are expected to increase the accuracy of genome-wide prediction and the power of genome-wide association analyses. However, genomic analyses of high-dimensional, highly correlated traits are challenging. We developed a method for implementing high-dimensional Bayesian multivariate regression to simultaneously analyze genetic variants underlying thousands of traits. As a demonstration, we implemented the BayesC prior in the R package MegaLMM. Applied to Genomic Prediction, MegaBayesC effectively integrated hyperspectral reflectance data from 620 hyperspectral wavelengths to improve the accuracy of genetic value prediction on grain yield in a wheat dataset. Applied to Genome-Wide Association Studies, we used simulations to show that MegaBayesC can accurately estimate the effect sizes of QTL across a range of genetic architectures and causes of correlations among traits. To apply MegaBayesC to a realistic scenario involving whole-genome marker data, we developed a 2-stage procedure involving a preliminary step of candidate marker selection prior to multivariate regression. We then used MegaBayesC to identify genetic associations with flowering time in *Arabidopsis thaliana*, leveraging expression data from 20,843 genes. MegaBayesC selected 15 single nucleotide polymorphisms as important for flowering time, with 13 located within 100 kb of known flowering-time related genes, a higher validation rate than achieved by a single-stage analysis using only the flowering time data itself. These results demonstrate that MegaBayesC can efficiently and effectively leverage high-dimensional phenotypes in genetic analyses.

## Introduction

The advent of high density genome-wide single nucleotide polymorphism (SNP) arrays in the past decades has provided exciting new material for the genetic analysis of complex traits. Linear mixed models that can integrate such large-scale genomic data are widely used for genomic prediction (Meuwissen *et al.* 2001; VanRaden 2008) and genome-wide association studies (Visscher *et al.* 2017). Recent advance in multi-omics methodologies now provide opportunities to generate large-scale transcriptomic, metabolomic, and epigenomic profiles as well. The integration of these high-dimensional phenotypes into association studies can increase power to detect causal variants. For example, gene expression profiling in thousands of genes has been used for the identification of genes that affect transcriptional variation (i.e. eQTLs) (Gibson and Weir 2005; McGraw *et al.* 2011), and integrative approaches combining genomic and gene expression data can have higher power to capture the true pathway associations underlying human diseases and complex traits (Xiong *et al.* 2012). In addition, recent developments of high-throughput phenotyping platforms have made the collection of thousands to millions of physiological measurements affordable to breeders (Araus *et al.* 2018). For example, images collected through thermal and hyperspectral cameras are used to increase the accuracy in genomic prediction for grain yield in wheat (Rutkoski *et al.* 2016). To further improve genomic prediction and to understand the underlying genetic mechanism, statistical models that enable the joint analysis of high-dimensional traits are required to establish the connection between phenomics and genomics.

Genomic analyses of high-dimensional, highly correlated data present analytic and computational challenges. The multivariate linear mixed model (MvLMM) is a widely used statistical model for the genetic analyses of 2 or more correlated traits (Henderson and Quaas 1976). However, most algorithms used to fit MvLMMs require repeated inversions of genetic and residual covariance matrices among all traits, with a computational burden that grows cubically to quintically as the number of traits increases (Zhou and Stephens 2014). MvLMMs are also susceptible to over-fitting unless sample sizes are very large. Re-parameterizing MvLMMs as Bayesian sparse factor models can alleviate much of this computational burden (Runcie and Mukherjee 2013; Runcie *et al.* 2021) and can significantly improve the accuracy of genomic prediction (Runcie *et al.* 2021). BSFG and MegaLMM are based on the assumption that the covariances among large sets of traits can be explained by a small set of latent factors (e.g. through gene regulatory networks), which is consistent with the discovery that variation in gene expressions of human diseases is mainly regulated by a few major disease-associated pathways (Xiong *et al.* 2012).

While MegaLMM addressed the statistical and computational challenges of applying MvLMMs to high-dimensional phenotypes, it permits a limited range of models for high-dimensional genotype data. Specifically, MegaLMM incorporates genomic data through 1 (or more) genomic relationship matrices, which imposes specific assumptions about the distribution and effect sizes of the underlying genetic variants, and does not allow direct inference on the identities of causal loci. Whole-genome regression methods (Meuwissen *et al.* 2001; Park and Casella 2008; Kizilkaya *et al.* 2010; Habier *et al.* 2011; Erbe *et al.* 2012; Cheng *et al.* 2015; Cheng, Kizilkaya, *et al.* 2018), on the other hand, encode a wide range of different and more flexible distributions on the effect sizes of causal genomic loci and allow for inference of the causal loci themselves. The Bayesian Alphabet methods in particular (Gianola *et al.* 2009; Kizilkaya *et al.* 2010; Habier *et al.* 2011; Gianola 2013; Moser *et al.* 2015; Wolc *et al.* 2016; Mehrban *et al.* 2017; Wang *et al.* 2020) use mixture priors on marker effects and are popular genetic models due to their incorporation of biologically meaningful assumptions and the variable selection procedure performed during model fitting. However, fitting Bayesian Alphabet methods to very large numbers of markers can also be computationally demanding even for a single trait, and extensions of these methods to multivariate traits are very limited.

In this paper, we develop a method for extending the Bayesian Alphabet whole-genome regression approaches to high-dimensional phenotypes using a Bayesian sparse factor model for applications in genome-wide prediction and genome-wide association studies. We focus on implementing the BayesC method in the R package MegaLMM as an example of a Bayesian Alphabet method (Kizilkaya *et al.* 2010; Habier *et al.* 2011; Cheng, Kizilkaya, *et al.* 2018), but extensions of MegaLMM to other priors should be straightforward. We call this model MegaBayesC, which is a specific parameterization and choice of prior distributions within the more general MegaLMM model family. We show that MegaBayesC can improve genomic prediction accuracy by leveraging mixture priors on marker effects and information from thousands of traits. In association studies with millions of markers, MegaBayesC is computationally demanding, but we propose a 2-step approach that can accurately estimate marker effects and improve power for association inference in both simulated and real data studies.

## Materials and methods

In a conventional MvLMM, the genetic and nongenetic correlations among t traits are modeled through 1 or more t×t genetic covariance matrices ($G_{m}$) and a t×t residual covariance matrix (R), respectively. The computational cost of fitting a MvLMM can be prohibitive when t is large due to the difficulty in taking inverses of the covariance matrices (Gilmour *et al.* 1995; Yang *et al.* 2011; Zhou and Stephens 2014). To overcome the computational challenge and overfitting in conventional MvLMMs, we reparameterized the conventional MvLMM as a factor model, as in Runcie *et al.* (2021), introducing K independent (unobserved) latent factors to account for the covariances among the t traits.

### Model description

In MegaLMM models, the variation among t observed traits is decomposed into 2 parts: the variation caused by dependencies on K independent latent factors, which induces correlations among the t observed traits, and the variation that is unique, or idiosyncratic, to each trait. In MegaBayesC, genetic values of latent factors are defined as a linear combination of marker effects, and BayesC priors (Meuwissen *et al.* 2001; Park and Casella 2008; Kizilkaya *et al.* 2010; Habier *et al.* 2011; Erbe *et al.* 2012; Cheng *et al.* 2015; Cheng, Kizilkaya, *et al.* 2018) are assigned to the marker effects. The model specification of MegaBayesC is described below

(1) $$Y=F\Lambda +X_{1}B_{1}+X_{2R}B_{2R}+E_{R}$$

with

(2) $$F=X_{2F}B_{2F}+E_{F}$$

where Y is an n×t matrix of observations for n individuals on t traits, F is an n×K matrix of latent factors for n individuals across K latent factors, and Λ is a K×t factor loading matrix whose elements ($\lambda_{kj}$) describe factor-trait relationships (e.g. the relationship between factor k and trait j). The K latent factors in F are further decomposed into genetic effects (i.e. $X_{2F}B_{2F}$) and residual effects (i.e. $E_{F}$) as shown in Equation (2). The genetic effects of latent factors are expressed as multiple regressions on genotype covariates, where $X_{2F}$ is an $n\times b_{2F}$ matrix of genotype covariates, and $B_{2F}$ is a $b_{2F}\times K$ matrix of marker effects for the K latent factors at $b_{2F}$ genotyped loci. $X_{1}$ is an $n\times b_{1}$ incidence matrix allocating the observations on t traits to $b_{1}$ fixed effects with coefficient matrix $B_{1}$. The residuals are similarly decomposed into trait-specific genetic effects (i.e. $X_{2R}B_{2R}$) and trait-specific residual effects (i.e. $E_{R}$), with $B_{2R}$ being a $b_{2R}\times t$ matrix of marker effects corresponding to the t traits at $b_{2R}$ genotyped loci. We expect users to generally choose identical design matrices for $X_{2R}$ and $X_{2F}$, allowing the same markers to affect the latent factors or trait residuals, but this is not required and we exclude $X_{2R}$ in some applications for computational efficiency.

If all sources of correlation among observed traits are explained by the latent factors, the residuals conditional on these factors become uncorrelated between different traits. Since the sources of correlation among observed traits are explained by independent latent factors, samples at each iteration of Markov chain Monte Carlo (MCMC) can be obtained simultaneously in parallel across traits and factors, which leads to significant reduction in the computational cost of model fitting.

### Prior specification

#### Genetic marker effects

Mixture priors are widely used for genetic marker effects in Bayesian regression methods in genome-enabled analysis. In this paper, the BayesC prior (Habier *et al.* 2011) is used for the marker effects (e.g. coefficients in $B_{2F}$). The BayesC mixture prior assumes that marker effects are independent and identically distributed, each of which has a point mass at zero with a marker exclusion probability π and follows a univariate normal distribution with a marker inclusion probability 1−π. For example, the prior distribution of the marker effect at locus i for the kth latent factor is shown as follows:

$$b_{{2F}_{k(i)}}=\left\{\begin{array}{cc} N(0,\sigma_{B2F_{k}}^{2}) & \text{probability}\;(1-\pi_{F_{k}})\\ 0 & \text{probability}\;(\pi_{F_{k}}) \end{array}\right\}$$

where $\sigma_{B2F_{k}}^{2}$ is the variance of marker effects corresponding to factor k. Due to the independence among latent factors and the independence among traits conditional on FΛ, marker effects can be efficiently sampled from a set of univariate BayesC models in parallel across traits and factors at each iteration of MCMC. We treat each marker exclusion probability for the K latent factors (e.g. $\pi_{F_{k}}$) and the t observed traits ($\pi_{R_{j}}$) as an independent unknown parameter to be estimated. Note that if marker inclusion probabilities for all factors were set to 1.0 (i.e. all markers are included), the model is equivalent to RR-BLUP, and we term this specific version of MegaLMM: MegaRRBLUP.

#### Factor loading matrix

The factor loading matrix (Λ) describes the relationship between latent factors and observed traits. Sparsity in this matrix implies that factors affect some, but not all traits, a key assumption in Bayesian sparse factor models (Carvalho *et al.* 2008). We use a BayesC mixture prior for the elements of Λ. For the factor loading that describes the relationship between factor k and trait j (i.e. $\lambda_{kj}$), its prior distribution is shown as follows:

(3) $$\begin{array}{rl} \lambda_{kj} & =\left\{\begin{array}{cc} N(0,\tau_{k}^{-1}\sigma_{R_{j}}^{2}) & \text{probability}\;(1-\pi_{\Lambda_{k}})\\ 0 & \text{probability}\;(\pi_{\Lambda_{k}}) \end{array}\right\}\\ \tau_{k} & =\prod_{h=1}^{k}\delta_{h}\\ \delta_{1} & =1\\ \delta_{h} & ∼\mathrm{Gamma}(a_{\delta },b_{\delta }),h=2\ldots K\\ \sigma_{R_{j}}^{2} & ∼\mathrm{Inv}\text{-}\mathrm{Gamma}(a_{\sigma },b_{\sigma }) \end{array}$$

The parameter $\tau_{k}$ stochastically increases from 1…K which has the effect of shrinking all values in the kth row of Λ toward zero. This means that all rows with row indices $k>k^{*}$ for some threshold $k^{*}$ will have all values close to zero and contribute a negligible amount to the total covariance. Therefore, as long as we choose $K>k^{*}$, the precise value of K is not important (Bhattacharya and Dunson 2011). In each run of MegaBayesC we check that the values in the last several rows of Λ contain only small values, implying that K is large enough. If this is not the case, we re-start the chain with a greater K. The only consequence of setting K larger than needed is an increase in the computation time required per iteration.

#### Other parameters

Prior distributions for all other parameters are the same as used in Runcie *et al.* (2021).

### Gibbs sampler

We use MCMC to sample from the posterior distributions of all parameters. The full conditional distributions of each Gibbs step are provided in the Supplementary File. The values of hyperparameters used for the analyses reported in this paper are provided in the Supplementary File. We used trace plots for the 5 largest elements in each of the K factors to assess convergence and ensure that the number of factors (K) is large enough to capture the covariance among observed traits. We also computed R-hat convergence diagnostics (Vehtari *et al.* 2021) for estimates of the genetic variance of focal trait using 3 MCMC chains. The genetic variance of the focal trait was computed as $X_{2F}B_{2F}\lambda_{f}$ (when $X_{2R}$ was excluded) with $\lambda_{f}$ being the column of Λ that specifies the relationship between factors and focal trait.

### Estimation of genetic values for genomic prediction

We assessed the performance of MegaBayesC as a tool for genomic prediction using hyperspectral data as additional traits to assist wheat yield prediction.

#### Data description

Best linear unbiased estimators (BLUEs) of grain yield and reflectances from 62 wavelength bands collected with an areal hyperspectral camera on each of 10 time-points during the growing season for 1,033 bread wheat lines were downloaded from CIMMYT Research Data (Krause *et al.* 2019). We analyzed results from the 2014–2015 breeding cycle under the Optimal Flat treatment. All lines were genotyped using the pipeline described in Poland *et al.* (2012). Markers with call rate ≤50% and minor allele frequency (MAF) ≤0.05 were removed. Missing genotypes were imputed by corresponding marker means. In our analysis, the 620 hyperspectral BLUEs were used as secondary traits (Runcie and Cheng 2019) to improve the prediction of the genetic value of grain yield, which is served as a focal trait in our prediction scenario. Both sets of traits were combined into a 1033×621 trait matrix Y.

#### Models

Five different models were used to predict the grain yield (GY): GBLUP_K, GBLUP_KH, MegaGBLUP, MegaRRBLUP, and MegaBayesC. These 5 models are described below.

##### GBLUP_K

A single-kernel single-trait model with a genomic relationship matrix, K, calculated from the full set of markers using the method of VanRaden (2008).

##### GBLUP_KH

A 2-kernel single-trait model with 2 relationship matrices: a genomic relationship matrix K and a hyperspectral reflectance relationship matrix H calculated as $H={SS}^{T}/620$ with S being the matrix of centered and standardized BLUEs of hyperspectral data. This method is analogous to the method proposed by Krause *et al.* (2019) as described in Runcie *et al.* (2021).

##### MegaGBLUP

This model implements a multivariate version of GBLUP_K as described in Runcie *et al.* (2021). The fixed effects $B_{1}$ included intercepts only. The random effects $B_{2R}$ and $B_{2F}$ were not included in the model. A random effect with covariance proportional to K was used to model the genetic relationships among lines. We ran MegaGBLUP with K=100 factors and assessed that the last several factors had all loadings close to zero.

##### MegaBayesC

The MegaBayesC model was specified as described above. $B_{1}$ included only intercepts, and $X_{2R}$ was not included for computational efficiency. Instead, to allow markers to directly affect grain yield (fitted as the first trait), we fixed the first factor to be a vector of 0’s, but with the value 1 as it’s first element. This latent factor thus only affects grain yield, so elements of the first column of $B_{2F}$ also only affect grain yield. For the remaining factors, the probability of an element being zero was considered as an unknown. The probability of a marker having a null effect on a latent factor was also considered as unknown for all the factors. We ran MegaBayesC with K=100 factors and assessed that the last several factors had all loadings close to zero. Estimated individual genetic merits of grain yield were computed as $u_{GY}=X_{2F}{\hat{B}}_{2F}{\hat{\lambda }}_{1}$, where ${\hat{\lambda }}_{1}$ denotes the first column of $\hat{\Lambda }$, which specifies the estimated relationship between all factors and grain yield.

##### MegaRRBLUP

The MegaRRBLUP model was identical to the MegaBayesC model except that the marker exclusion probability $\pi_{F_{k}}$ was set to 0 so all markers were included. This model should be identical to MegaGBLUP except that the prior distribution of Λ is a BayesC prior (with factor loading exclusion probability $\pi_{\Lambda_{k}}$ estimated from the data) in MegaRRBLUP, which differs slightly from the Bayesian Horseshoe prior used in MegaGBLUP (Runcie *et al.* 2021).

All Bayesian methods were run for 10,000 iterations, with 2,000 used as burn-in and a thinning rate of 2.

#### Cross validation

We used cross-validation to evaluate the predictive performance of different models by masking the grain yield of randomly selected subsets of 50% of the lines during model fitting and comparing the predictions of the genetic values of lines with masked values to their observed grain yield. Since we did not mask the hyperspectral data from these validation individuals to use those secondary traits as additional features for genetic value predictions, estimating prediction accuracy using Pearson’s correlation between predicted and observed grain yield values will be biased (Runcie and Cheng 2019). Instead, we estimated the accuracy as the genetic correlation corrected by grain yield heritability (Daetwyler *et al.* 2013; Runcie and Cheng 2019) as implemented in Runcie *et al.* (2021). The cross-validation process was repeated 20 times with different selections of masked lines. Since these cross-validation repetitions re-use validation data, estimates of genetic prediction accuracy across repetitions are correlated and t-tests of differences among models become anti-conservative. We used a modified t-statistic to account for this correlation when comparing across methods: $t=(\frac{1}{20}\sum_{\;j=1}^{20}d_{j})/\sqrt{(\frac{1}{20}+\frac{n_{2}}{n_{1}}){\hat{\sigma }}^{2}}$, where $n_{1}$ and $n_{2}$ are the sizes of the training and testing sets for the focal trait, respectively (Bouckaert and Frank 2004).

### Estimation of marker effects for association inference

We conducted 2 simulation studies and 1 real data analysis to investigate the ability of MegaBayesC to accurately estimate marker effects. First, we simulated a population with independent and uncorrelated SNPs to assess the ability of MegaBayesC to distinguish between genetic and nongenetic sources of variation in a focal trait by using information from correlated traits. Second, we designed a simulation study using marker data from a real *Arabidopsis thaliana* population to study the effects of population structure and linkage among markers. Finally, we used MegaBayesC to model real flowering time phenotypes from this Arabidopsis population and assessed whether expression data from 20,843 genes could improve gene discovery by genome-wide association study (GWAS).

Although MegaBayesC greatly reduces the computational burden of Bayesian multivariate regressions by decomposing phenotypic covariances into a set of K laten factors, whole-genome regression models with hundreds of thousands of candidate markers are computationally prohibitive. In our second simulation study and our real data analysis, we implemented a 2-stage approach for BayesC-based methods. In the first stage (i.e. the preselection stage), we selected a small proportion of SNPs to take forward into a full BayesC analysis by running a single-trait GWAS on individuals that have no observation on secondary traits. We used LD-based clumping to select thousands of potentially important SNPs. After the preselection stage, the records of focal and secondary traits from the remaining individuals were analyzed in BayesC-based methods using only the preselected potentially important SNPs.

#### Simulated study in a population without structure or linkage disequilibrium

We created a simulated population of n=3,000 individuals and p=2,000 independent SNPs. An n×p matrix of genotypic covariates was generated by random sampling from {0,1,2}. We then created simulated phenotypic data for a single focal trait and many correlated “secondary” traits. The performance of MegaBayesC was compared to a single-trait BayesC model (ST-BayesC) based on the accuracy of estimated marker effects for the focal trait. We induced genetic and nongenetic covariation among the traits through latent factors. The majority of variance in the focal trait was attributed to the latent factors. In Scenario 1, we created latent factors whose variation was primarily determined by the genetic markers (i.e. high-heritability latent factors), and in Scenario 2, the latent factors were predominantly nongenetic (i.e. low-heritability factors).

We studied 4 parameters that we expected to influence the relative performance of MegaBayesC and ST-BayesC: (1) the number of latent factors ($n_{\;factor}$), (2) the number of correlated traits ($n_{trait/factor}$) controlled by each factor, (3) the number of QTL (i.e. causal variants) that control each factor ($n_{qtl/factor}$), and (4) the heritability of the factors. In this simulation study, $n_{\;factor}=\{2,6,9\}$, $n_{trait/factor}=\{2,20\}$, $n_{qtl/factor}=\{10,20,30\}$, and 2 heritable patterns of latent factors were considered.

To generate simulated phenotype data, we first used $n_{\;factor}$ and $n_{trait/factor}$ to construct a factor loading matrix (Λ). For example, when $n_{trait/factor}=2$ and $n_{\;factor}=2$, 4 (i.e. $n_{\;factor}\times n_{trait/factor}$) observed traits were simulated, with 2 different observed traits linked to each factor. Since the first observed trait was treated as the focal trait, and all factors were assumed to contribute to its variation, factor loadings in the first column of Λ were set to 1. To minimize the complexity of this simulation, all the nonzero factor loadings in Λ were set to 1. Therefore, the simulated Λ given $n_{trait/factor}=2$ and $n_{\;factor}=2$ was

$$\Lambda =\left[\begin{array}{cccc} 1 & 1 & 0 & 0\\ 1 & 0 & 1 & 1 \end{array}\right]$$

By constructing Λ in this way, all factors except the first were linked to $n_{trait/factor}+1=3$ observed traits, while the first factor was linked to $n_{trait/factor}=2$ observed traits. A similarly structured Λ was constructed for other combinations of $n_{trait/factor}$ and $n_{\;factor}$.

After defining Λ, genetic variation controlled by QTL and nongenetic residual variation in each factor was simulated. $n_{qtl/factor}$ QTL were selected for each factor, and Gaussian variation was added such that the variance explained by QTL was a specific percentage of the total variance in the factor. In Scenario 1, QTL accounted for 95% of the variance of each factor (i.e. $\sigma_{FG}^{2}/(\sigma_{FG}^{2}+\sigma_{FE}^{2})=0.95$ with $\sigma_{FG}^{2}$ being the genetic variance of factors and $\sigma_{FE}^{2}$ being the residual variance of factors). In Scenario 2, only the first factor was associated with QTL (again with 95% of its variance explained by the QTL), and the remaining factors had independent variation. Finally, trait-specific variation was added to each trait, accounting for approximately 10% of its total variance.

As a consequence of these simulation choices, the 2 scenarios differed in several key aspects of the genetic architecture and correlation structures between the focal trait and the secondary traits. In Scenario 1, all factors were controlled predominately by genetic variation and every QTL for every factor was indirectly a QTL for the focal trait, and all secondary traits had strong genetic correlations with the focal trait. In Scenario 2, most factors were controlled by nongenetic variation; only the first factor was controlled by QTL. Therefore, all secondary traits were phenotypically correlated with the focal trait, but most of these correlations were nongenetic.

In both scenarios, as $n_{\;factor}$ and/or $n_{qtl/factor}$ increased, the magnitude of variation explained by each QTL decreased to hold the total percentage of variation in the focal trait explained by QTL constant.

In Scenario 1, when $n_{\;factor}=9$ and $n_{qtl/factor}=10$, the 90 QTL each explained ≈0.97% of the total variance (Fig. 3). As $n_{qtl/factor}$ increased to 30, the number of QTL for the focal trait increased to 270 and each accounted for around 0.29% of the total variance of focal trait. The percentage of variance explained by QTL was constant across values of $n_{\;factor}$. In this scenario, all QTL for all factors contributed to the variation in the focal trait. For example, when $n_{\;factor}=6$ and $n_{qtl/factor}=10$, each factor was influenced by 10 QTL, for a total of 60 QTL. During this process, some SNPs were stochastically selected more than once, and thus some QTL had effects on more than 1 factor.

In Scenario 2, as $n_{\;factor}$ increased, the proportion of variance explained by QTL decreased. For example, when $n_{\;factor}=6$, the genetic variance accounted for 14% of the total variance of focal trait. With $n_{\;factor}=9$, the percent of variance explained by genetic markers decreased to 9%. Furthermore, when $n_{\;factor}=9$, the variance explained by each marker decreased from 0.90% to 0.31% as the number of QTL increased from 10 to 30. For a given $n_{\;factor}$ and $n_{qtl/factor}$ the per-QTL effect sizes in both scenarios were comparable, but since there were more genetic factors in Scenario 1, the total variance in the focal trait controlled by QTL was larger.

Based on the combination of $n_{trait/factor}$, $n_{\;factor}$, $n_{qtl/factor}$, and the heritable patterns, a total of 3×3×2×2 conditions were studied in this simulation study. Ten replicates were conducted for each of the 36 conditions.

When fitting models to these simulated data, we included the intercept for each trait as the only fixed effect. We compared MegaBayesC to a single-trait implementation of BayesC (ST-BayesC) using the JWAS package (Cheng, Fernando, *et al.* 2018). The model specification of MegaBayesC was similar to that used in the **Genomic Prediction** application, except: (1) no fixed factor loadings were included in Λ, (2) Λ the number of factors fitted in the model was K=10. We estimated the total marker effects on the focal trait as $\alpha_{f}=B_{2F}\lambda_{1}$, where $B_{2F}$ is the matrix of marker effects of latent factors and $\lambda_{1}$ denotes the first column of Λ specifying the relationship between factors and focal trait, i.e. summing the QTL effects on the latent factors weighted by the relationships between each factor and the focal trait. Both models were run for 10,000 iterations, with a burn-in of 2,000 iterations and a thinning rate of 10. We measured the performance of each method (MegaBayesC and ST-BayesC) by calculating the square root of the mean square error (RMSE) of estimated marker effects.

#### Simulation study in a real Arabidopsis population

We created a second set of simulated datasets based on real genotypes from 1003 Arabidopsis thaliana accessions. Genotype data were downloaded from the 1,001 genomes project (Alonso-Blanco *et al.* 2016). In a real population, the presence of linkage disequilibrium (LD) between loci and variable allele frequencies among markers increase the complexity of genetic association analyses. We removed SNPs with MAF ≤0.05 and missing genotype rate ≥0.1 using PLINK 1.9 (Purcell *et al.* 2007), leaving 802,427 variants used for downstream analysis.

To ensure the QTL were independent, we pruned SNPs with an LD threshold of 0.8 in windows of 500 SNPs, using a sliding window of 100 SNPs. We randomly selected 20 QTL from these SNPs, and generated 10 latent factors, each affected by 2 different QTL. In this simulation, the structure of the variance of the focal trait was simplified. All genetic variance in all traits was driven by the QTL effects on the latent factors, while all nongenetic variance was trait-specific. In this way, the observed traits (Y) was expressed as $Y=X_{2}B_{2F}\Lambda +E_{R}$.

We set each element of the first column of Λ to 0.5 so that all 10 of the factors contributed equally to the focal trait. Each factor was additionally linked to 20 different secondary traits with factor loadings equal to 1. Other elements in Λ were set to be 0. Therefore, a total of 201 traits were simulated.

The proportion of genetic variance in the focal trait was set to ≈60% (i.e. $h_{\;focal}^{2}=0.6$). To ensure that the variance explained by each QTL was consistent (≈1–5% of the total variance), QTL effects were sampled from a uniform distribution U(3,5), and a randomly chosen half of those effects were multiplied by −1. In addition, since the heritability of secondary traits such as gene expression is often higher than that of focal trait in real data applications, the heritabilities of the 200 secondary traits were each set to be 0.8.

Finally, to parallel our real data analysis below, secondary trait data were simulated for only 649 of the 1003 Arabidopsis accessions. The 354 remaining accessions had records for only the focal trait. Hundred replicates were conducted in this simulation study.

After creating the simulated data, we applied 3 methods to identify QTL and estimate their effects on the focal trait: (1) single-trait Genome-Wide Association Studies (GWAS) using GCTA (Yang *et al.* 2011) (ST-GCTA); (2) single-trait BayesC implemented in JWAS (Cheng, Fernando, *et al.* 2018) (ST-BayesC); and (3) MegaBayesC implemented in MegaLMM.

A 2-stage analysis as described above was implemented for ST-BayesC and MegaBayesC. In the first stage, a single-trait GWAS on the 354 individuals without records on secondary traits was implemented in GCTA. After running the GWAS, we sorted SNPs by P-value, removed SNPs with P-values larger than 0.01, then used a greedy algorithm to select the most-significant SNPs and mask all nearby SNPs (within 250 kb) with $r^{2}>0.5$ (Purcell *et al.* 2007). After the preselection stage, the records of focal and secondary traits from the remaining 649 individuals were analyzed in ST-BayesC and MegaBayesC using only the preselected potentially important SNPs.

The model specification of MegaBayesC was similar to that used in the previous simulation study for the independent populations, except we set K=30. In MegaBayesC, the total marker effects of the focal trait were computed as $\alpha_{f}=B_{2F}\lambda_{f}$, where $B_{2F}$ is a $b_{2F}\times K$ matrix of marker effects for latent factors, with $b_{2F}$ being the number of SNPs selected at the preselection stage and $\lambda_{f}$ being the column of Λ that specifies the relationship between factors and focal trait. Furthermore, to demonstrate that the improved performance of MegaBayesC is attributed to not only the use of the BayesC prior on the marker effects but also the utilization of information from correlated secondary traits, a ST-BayesC analysis was also performed for the 649 individuals at the second stage. MCMC chains of 50,000 iterations were run for the BayesC-based methods with the first 10,000 iterations discarded as burn-in. We used a thinning rate of 1 sampler per 100 iterations to compute posterior means of model parameters.

In addition to the 2-stage analysis, a 1-stage ST-GCTA was performed using the whole-genome SNP information and the phenotypes of the focal trait from all 1003 individuals. To compare with the 2-stage analysis, the selection of potentially important SNPs was done based on the 1-stage ST-GCTA result in the same manner as that in the preselection stage.

**Fig. 1.**
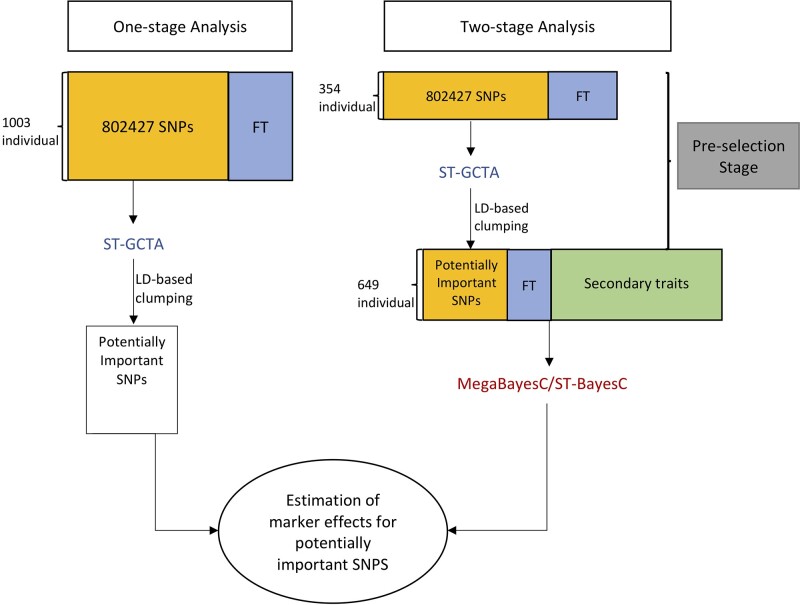
Graphical representation for the procedure of 1-stage and 2-stage analyses performed for the estimation of marker effects. FT represents the focal trait, ST-GCTA represents the single-trait GWAS implemented in GCTA, and ST-BayesC represents the single-trait BayesC method. In ST-BayesC, only phenotypes of FT and genotypes of the preselected potentially important SNPs were used.

Figure 1 shows the procedures performed to estimate marker effects in the 3 different methods.

RMSEs of the QTL effect sizes and the variance in the focal trait explained by the QTL were used to evaluate the accuracy of estimation of QTL effects by different methods. The variance explained by marker l was computed as $var(\alpha_{lf}x_{l})$, where $\alpha_{lf}$ is the marker effect of SNP l on focal trait, and $x_{l}$ is the vector of genotypic covariates for SNP l. To score QTL accuracy, we parsed the detected QTL in 3 ways: (1) If the true QTL were selected in the set of potentially important SNPs (e.g. Stage 1), the estimated effects were compared directly to the true effects. (2) If a SNP in imperfect LD with the true QTL was selected instead of the true QTL, we flagged its estimated effect size in the accuracy comparison because the incomplete linkage and different allele frequencies of the 2 SNPs mean that the estimated effect size will not be directly comparable to that of the true QTL. However, the variance attributed to the marker should be similar to the true QTL as long as $r^{2}$ is high. (3) If neither the true QTL nor any of its linked SNPs was selected in the potentially important SNPs, we set the estimated marker effect and variance explained by this QTL to 0. For the purpose of unit consistency, RMSE of estimated marker effects and estimated marker-explained standard deviation (i.e. square root of marker-explained variance) across different methods were compared. In this study, a SNP was considered to be linked to a QTL if the squared correlation between its genotypic covariate and the QTL genotype was greater than 0.4.

#### Genetic association analysis of *Arabidopsis thaliana* flowering time and gene expression

Phenotypes of flowering time from 1,003 accessions and expression data of 20,843 genes from 649 accessions were used, with flowering time selected as our focal trait. Gene expression data were downloaded from NCBI GEO (Barrett *et al.* 2012). Genes with average counts smaller than 10 were removed and the remaining gene counts were normalized and variance stabilized as per Runcie *et al.* (2021) using DESeq2 (Love *et al.* 2014). The 2-stage MegaBayesC and 1-stage ST-GCTA analyses described above were performed again on this dataset. LD-based clumping was done to select potentially important SNPs for both methods. The model specification of MegaBayesC was similar to that used in the **Genomic Prediction** application. A MCMC chain of 80,000 was run with the first 20,000 iterations discarded as burn-in. We used a thinning rate of 1 sample per 100 iterations to compute posterior means of model parameters. In the 2-stage MegaBayesC analysis, potentially important SNPs with explained proportion of variance >0.1% were classified as significant SNPs, while in the 1-stage ST-GCTA analysis, potentially important SNPs with P-value $<1\times 10^{-5}$ were classified as significant SNPs. We compared each significant SNP to a list of genes previously known to influence flowering time in Arabidopsis (Bouché *et al.* 2016), and counted as a match (i.e. a true positive hit) if a SNP was within ±100 kb distance from at least 1 of the reported genes. Otherwise the significant SNP was conservatively considered as a false positive association.

## Results

### MegaBayesC and MegaRRBLUP achieve similar prediction accuracy as MegaGBLUP

We tested if whole-genome regression models could match or exceed the performance of univariate genomic prediction (GBLUP) and multivariate genomic prediction (MegaGBLUP) using data from a breeding trial of bread wheat. We compared the genomic value prediction accuracy of MegaBayesC and MegaRRBLUP to MegaGBLUP in this dataset, where we leveraged 620 hyperspectral phenotypes measured on 1,033 bread wheat lines to supplement genotype-based predictions of genomic value for grain yield. As baseline, we performed univariate genomic prediction using either the marker-based genomic relationship matrix (GBLUP_K) or a 2-kernel univariate model using both a genomic relationship matrix and a hyperspectral reflectance relationship matrix (GBLUP_KH). Prediction accuracy was assessed by cross-validation where for each of 20 replicates, grain yield values of 50% of the lines were masked and used as an independent testing set. Estimated genetic correlations between predicted and observed yields in the testing set were used as the cross-validation statistic.

**Fig. 2.**
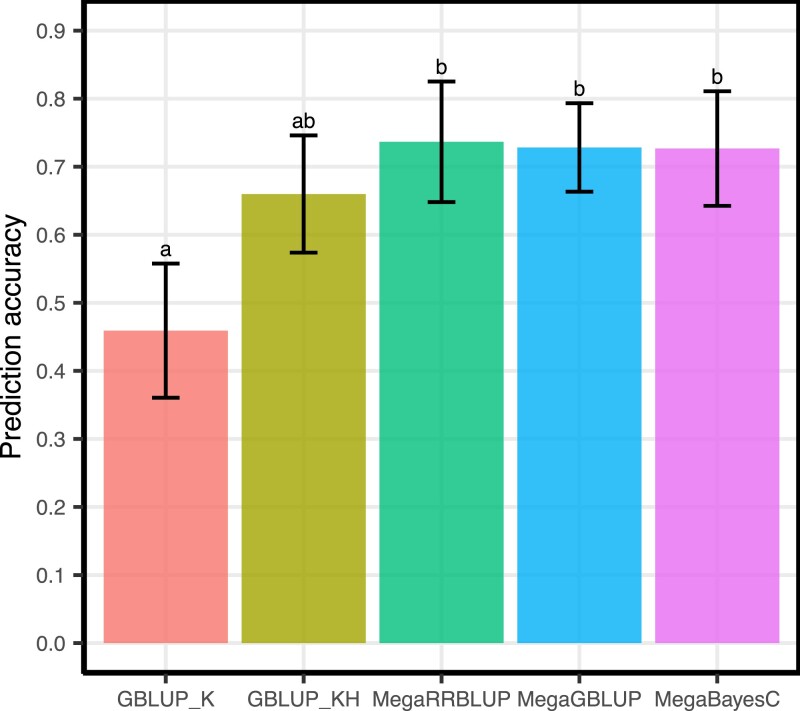
Genomic value prediction performance of 5 models for wheat yield. Records of yield, 620 hyperspectral phenotypes, and genotype data for 1,033 lines were used. Bars show the mean prediction accuracy (± adjusted standard error) for each model, and letters show the statistical significance of mean difference between methods based on an adjusted t-statistics that accounts for correlations between samples.

As shown in Fig. 2, the single-kernel GBLUP_K model had the lowest average prediction accuracy across cross-validation runs (r=0.46) in this dataset, followed by the 2 kernel, but still univariate GBLUP_KH model (r=0.66). MegaBayesC achieved a higher average prediction accuracy (r=0.73) to the univariate models, but a similar average accuracy to MegaRRBLUP and MegaGBLUP (r=0.74 and 0.73, respectively). However, because our cross-validation runs re-used observations (due to the necessary 50:50 training split to estimate genetic prediction accuracy using the method of Runcie and Cheng 2019), the differences in estimated accuracy among the 4 methods incorporating the hyperspectral data were not significant at α=0.05 using a re-sampling-adjusted t-statistic: $t=(\frac{1}{20}\sum_{\;j=1}^{20}d_{j})/\sqrt{(\frac{1}{20}+1){\hat{\sigma }}^{2}}$ (Bouckaert and Frank 2004). These results indicate that MegaBayesC can effectively leverage multivariate associations for genomic prediction, but there was no benefit from the more complex MegaBayesC model relative to the simpler MegaGBLUP model.

**Fig. 3.**
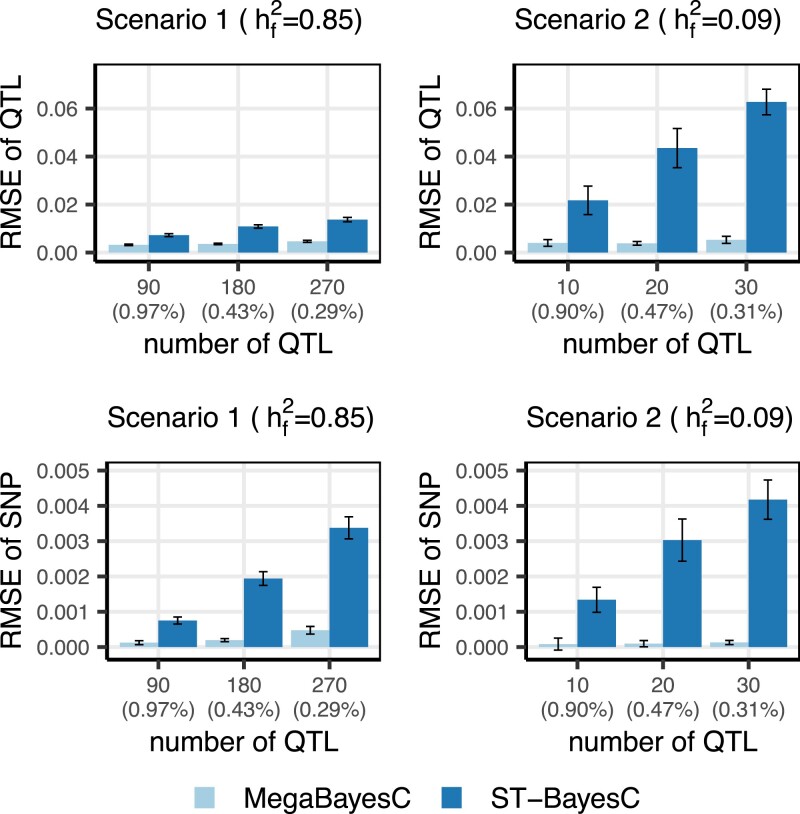
RMSE of estimated QTL effects and SNP effects, respectively, under 2 scenarios. The upper panels show RMSE of estimated QTL effects under 2 scenarios. The lower panels show RMSE of estimated SNP effects under 2 scenarios. The left panels show RMSE for Scenario 1, where all latent factors had high heritability ($h^{2}=0.95$). The right panels show RMSE for Scenario 2, where only 1 of the factors had high heritability (i.e. factor 1 had $h^{2}=0.95$ and the remainder factors had $h^{2}=0$). Results are shown for the simulation setting with $n_{trait/factor}=2$ and $n_{\;factor}=9$. The average proportion of total variance explained by 1 QTL was shown in the parenthesis of x-axis label.

### MegaBayesC improves estimation of marker effects in simulated populations with independent markers

Next, we ran a set of simulations to evaluate the ability of MegaBayesC to identify and accurately estimate the effect sizes of genetic variants for a set of correlated traits under different genetic architectures. Specifically, we tested whether MegaBayesC improved the estimation of variant effect sizes of a single focal trait when phenotypes of other correlated traits (i.e. secondary traits) were provided.

Since the magnitude and causes (genetic vs. nongenetic) of the covariance structures among traits determine the usefulness of the secondary traits, we considered 2 covariance structures. In both cases, we began by simulating a set of latent factors partially controlled by genetic variation. In Scenario 1, the majority of variation in the focal trait was controlled by latent factors that were dominated by genetic variation. In Scenario 2, the majority of variation in the focal trait was controlled by latent factors dominated by nongenetic sources of variation. We compared the estimation of marker effects between ST-BayesC (which ignored all secondary traits) and MegaBayesC (which used all trait data at once). We scored the accuracy of each method by the RMSE of estimated marker effects. In both scenarios, as the genetic architecture increased in complexity (i.e. the number of QTL increased and the average size of each QTL decreased to keep the total percentage of variation attributable to the QTL constant), the performance of ST-BayesC decreased (RMSE increased) much more dramatically than MegaBayesC. Figure 3 shows RMSE of estimated effects for QTL and SNP, respectively, (i.e. QTL are markers with a nonzero effect and SNPs are markers with a true effect size of zero) under the 2 scenarios for the simulation setting where the largest difference of RMSE was observed between MegaBayesC and ST-BayesC, with $n_{trait/factor}=2$ and $n_{\;factor}=9$. Results for other combinations of $n_{\;factor}$, $n_{trait/factor}$, and $n_{qtl/factor}$ are shown in the Supplementary File (Supplementary Fig. S1). In Scenario 1, the number of latent factors had no direct effect on the performance of ST-BayesC beyond its effect on the number of QTL. Also, the number of traits linked to each factor (i.e. $n_{trait/factor}$) did not significantly affect the performance of MegaBayesC in both Scenario 1 and Scenario 2. This shows the ability of MegaBayesC to capture the underlying sources of correlations among traits by optimizing the utilization of secondary traits, even when each factor only has 1 linked secondary trait included in the model.

For ST-BayesC, the RMSE of estimated marker effects increased significantly as marker-explained variances decreased in both scenarios. Compared to Scenario 1, the increase of RMSE for estimated effects of QTL was greater in Scenario 2, while the increase of RMSE for estimated effects of SNPs were similar between the 2 scenarios. This indicates that the performance of ST-BayesC to identify QTL was affected by the marker-explained variance as well as the variance structure of the focal trait.

In contrast, the performance of MegaBayesC was relatively constant across scenarios as measured by RMSE. In terms of the estimation of effect sizes of QTL, the influence of the variance structure and the marker-explained variance was negligible, which led to a relatively constant RMSE across the simulation settings. At the same time, MegaBayesC was able to shrink most SNPs more effectively toward zero, especially in Scenario 2, when the ratio of number of QTL to number of SNPs was smaller.

**Fig. 4.**
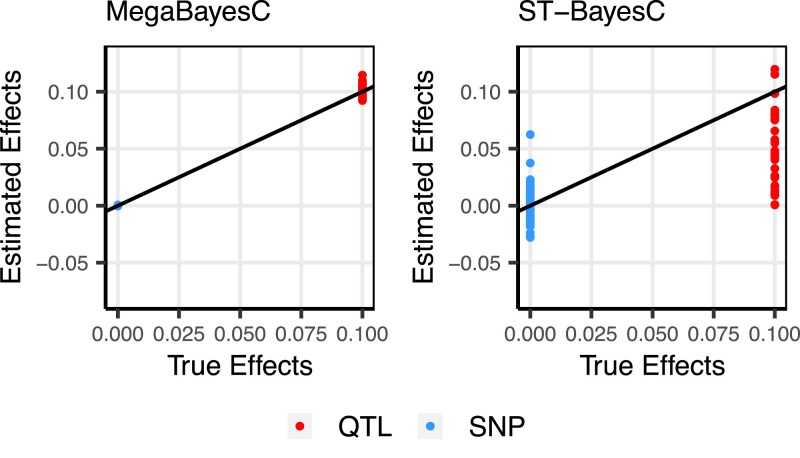
Scatter plot of estimated marker effects versus true marker effects for the simulation setting with $n_{\;factor}=9$, $n_{qtl/factor}=30$, and $n_{trait}=2$ in Scenario 2, where all factors have effects on the focal trait but only 1 of them is a genetic factor (i.e. $h^{2}>0$). Red and blue colors specify QTLs (True effect size =0.1) and SNPs (True effect size = 0), respectively. The solid line represents the line y=x.

To further explore the differences in the performance of ST-BayesC and MegaBayesC in Scenario 2, we plotted the estimated marker effects under 1 example simulation with $n_{\;factor}=9$, $n_{qtl/factor}=30$, and $n_{trait}=2$ (Fig. 4).

For ST-BayesC, some QTL were successfully selected by the model and their effect sizes were accurately estimated close to the true value of 0.1. However, for the majority of QTL, the estimated marker effects were shrunk toward 0s. On the other hand, ST-BayesC erroneously estimated effect sizes of null-effect SNPs from −0.03 to 0.06. In contrast, the marker effects of QTL and null-effect SNPs were accurately estimated by MegaBayesC (Fig. 4).

### Estimation of explained variance of markers in a population simulated using real genotype data

To explore the ability of MegaBayesC to accurately identify QTL and estimate their effect sizes in the presence of LD, we generated simulated phenotypes based on real genotypes from an Arabidopsis population. We then ran association analyses using 3 methods: The direct (i.e. 1-stage) method, ST-GCTA, that only uses the focal trait, and 2 2-stage methods: ST-BayesC and MegaBayesC, which both rely on a preselection stage to select a set of candidate SNPs using 1 partition of the population, and then an assay stage where the effects of those SNPs on the focal trait are modeled in the second partition of the population. We compared the performance of the models by the RMSE of estimated marker effects and marker-explained variances.

**Fig. 5.**
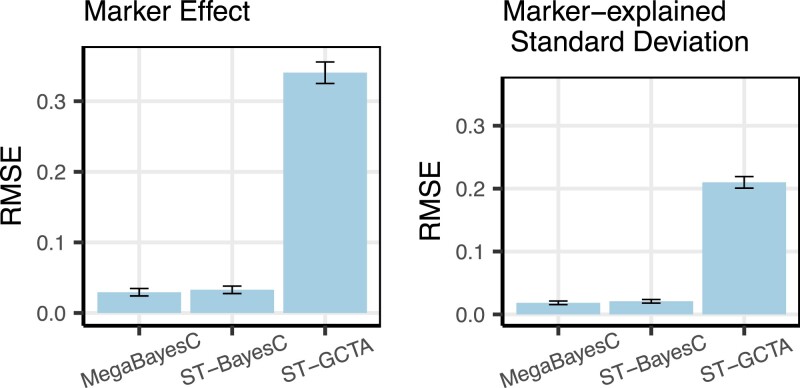
RMSE of estimated marker effects and estimated marker-explained standard deviations (i.e. square root of marker-explained variances) across different methods. The performance of 2-stage (ST-BayesC and MegaBayesC) methods and 1-stage (ST-GCTA) method was compared.

Figure 5 shows the RMSE of estimated marker effects and estimated marker-explained standard deviations from the simulated phenotype data. The 2-stage MegaBayesC method achieved the lowest RMSE for both marker effects and marker-explained standard deviations, followed by the 2-stage analysis incorporating ST-BayesC, and then the 1-stage single-trait GWAS (ST-GCTA). The RMSE of the 1-stage single-trait GWAS was around 10 times larger than that of the 2-stage BayesC-based analyses, while the difference between ST-BayesC and MegaBayesC was much smaller. Furthermore, the RMSE of estimated marker-explained standard deviations was generally lower than that of estimated marker effects. The larger RMSE of estimated marker effects is likely due to the selection of linked SNPs rather than the true causal QTL in the preselection stage.

**Fig. 6.**
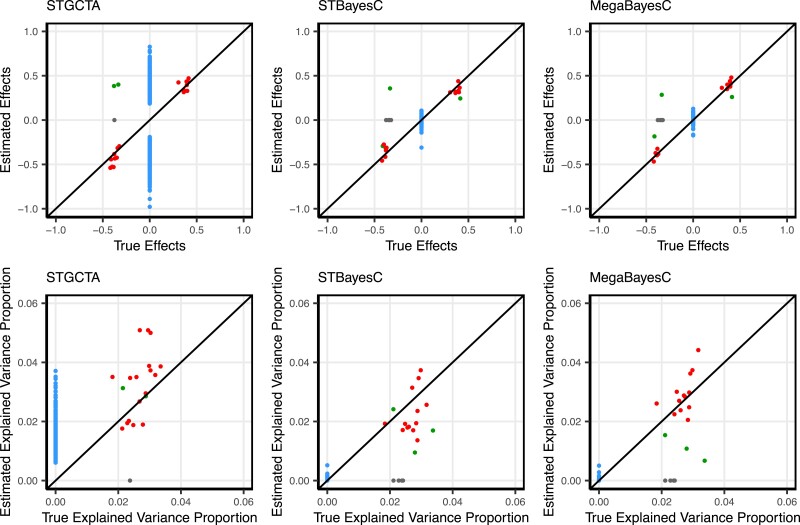
The relationship between estimated and true values of marker effects and marker-explained proportion of variance for focal trait. Three different methods (ST-GCTA, ST-BayesC, and MegaBayesC) were compared. Details of each method are presented in “Materials and methods”. Red and grey points indicate markers that are causal QTL. Red markers were selected by the first-stage analysis but grey markers were not and so have an estimated effect size of zero. Green and blue points indicate markers that are not causal QTL but were selected by the first-stage analysis. Green markers are in strong LD with true causal QTL but blue markers are not.

To further explore the difference in the performance of ST-BayesC and MegaBayesC in this simulation scenario, we present the relationship between true and estimated marker effects for 1 replicate in Fig. 6. In this simulation, 19/20 true causal QTL were selected by ST-GCTA, and only 16 were selected in the preselection stage for the 2-stage methods, ST-BayesC and MegaBayesC. In all 3 cases, the effect sizes of these selected QTL were accurately estimated. However, the effect sizes of many null-effect SNPs were dramatically overestimated by ST-GCTA, leading to an overall high false positive rate. In contrast, although a few true causal QTL were missed in the preselection stage, SNPs with null effects that were moved forward into stage 2 were estimated to have very small effects by both ST-BayesC and MegaBayesC.

Note that in some cases, SNPs that are in LD with true QTL were selected instead of the causal QTL. When the linkage phase was negative, the estimated effect sizes for linked SNPs have the opposite sign, which increases the reported RMSE. However, even in these cases, the proportion of variance explained by these linked markers is close to the proportion that would have been explained by the true QTL, so the effect of LD on the RMSE of marker-explained variances is minimized.

### Identifying candidate genes for flowering time in Arabidopsis using gene expression data as secondary traits

We applied the 2-stage MegaBayesC and the 1-stage single-trait GWAS (ST-GCTA) to the task of identifying candidate genes that regulate flowering time in *Arabidopsis thaliana* using actual flowering time measurements and genotype data from 1003 *A. thaliana* accessions. In MegaBayesC, we included the expression of 20,843 genes measured on 649 of the accessions as secondary traits.

**Fig. 7.**
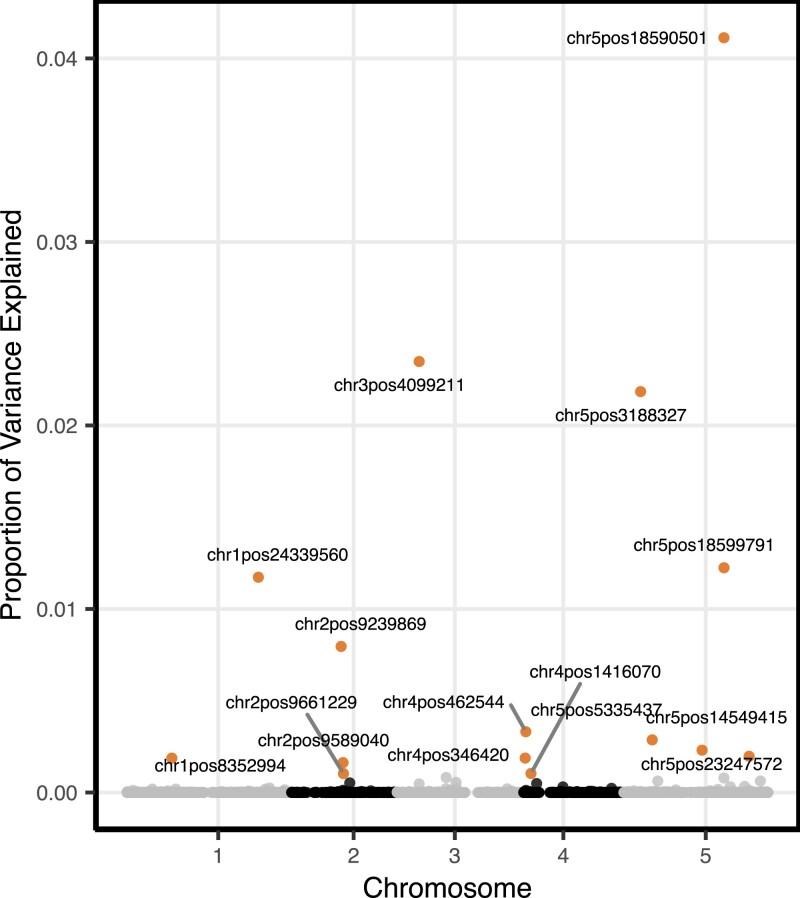
Marker-explained proportion of variance for potentially important SNPs by the 2-stage analysis using MegaBayesC. The top 15 SNPs that explained the greatest proportions of variance in flowering time are highlighted.

**Fig. 8.**
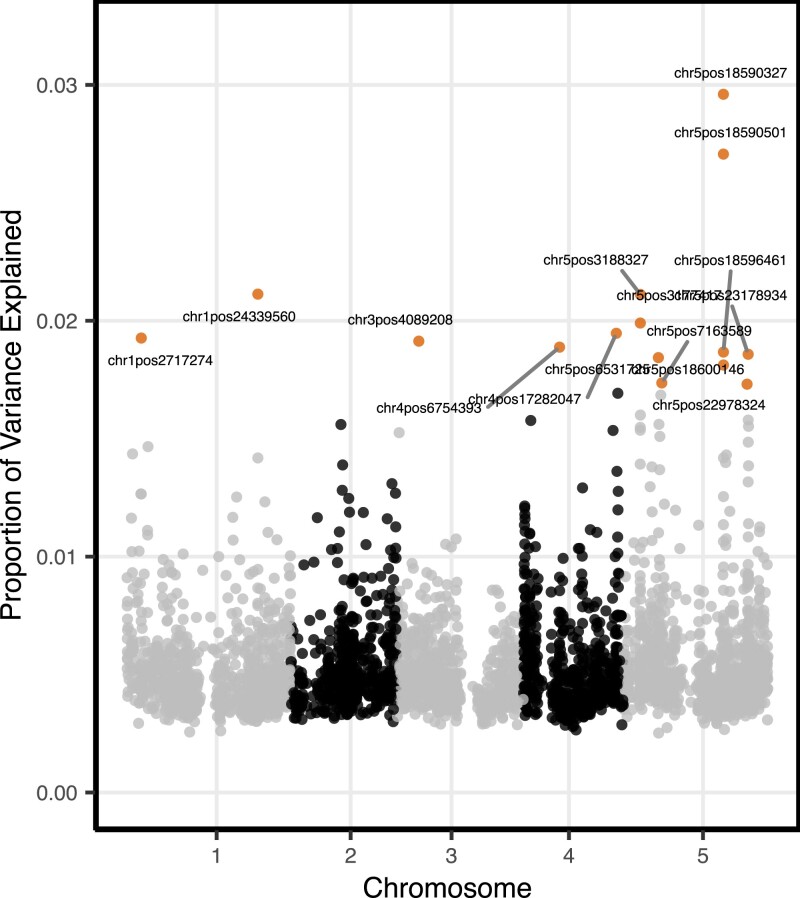
Marker-explained proportion of variance for potentially important SNPs by the 1-stage ST-GCTA analysis. The top 15 SNPs that explained the greatest proportions of variance in flowering time are highlighted.

Potentially important SNPs with marker-explained variance proportion greater than 0.1% in MegaBayesC and potentially important SNPs with P-value smaller than $10^{-5}$ in ST-GCTA were selected as significant SNPs. MegaBayesC was better able to select a limited number of candidate SNPs based on per-marker variance explained (Fig. 7) than ST-GCTA (Fig. 8) by shrinking the vast majority of SNP effects to zero.

We assessed the accuracy of these associations by checking whether known flowering time-related genes are located near to the SNPs selected by each model.

Using MegaBayesC, we selected 15 significant SNPs and 13 of these were located within 100 kb of known flowering time-related genes. Note that these known genes were generally not the nearest gene to the significant SNPs, but associations at this distance are not uncommon in Arabidopsis (Sasaki *et al.* 2021). In some cases, more than 1 gene was within ±100 kb of a significant SNP, so a total of 15 known flowering-time-related genes were within 100 kb of the 13 significant SNPs. For ST-GCTA, we selected 34 significant SNPs, among which 26 SNPs were located within 100 kb of known flowering time-related genes. In several cases, more than 1 selected SNP was within 100 kb of a known flowering-time related gene, so a total of 15 known flowering-time-related genes were within 100 kb of the 26 significant SNPs. Detailed comparison on detected genes between MegaBayesC and ST-GCTA is shown in Table 1.

**Table 1.** Detailed information on detected genes from ST-GCTA and MegaBayesC.

Method	Number of significant SNPs	Number of false positives	Detected genes
MegaBayesC	15	2	AGL17, **FLC**, **FT**, FRI, **FRL1**,
			GRP7, **LIF2**, MED18, **AtNDX**, **PIE1**,
			SEF, SVP, **VIN3**, **ZTL**, and **DOG1**
ST-GCTA	34	8	CIB2, **FLC**, **FT**, **FRL1**, JMJ14,
			LATE, **LIF2**, MRG1, **AtNDX**, **PIE1**,
			PRMT4A, TSF, **VIN3**, **ZTL**, and **DOG1**

Bold fonts are used to indicate genes that are detected in both methods.

## Discussion

The emergence of new types of phenotype data, such as gene expression or spectral reflectances, has created a demand for the development of robust models that are able to analyze large numbers of phenotypes in genome-enabled analysis. Although Bayesian regression models with mixture priors allow for more biologically meaningful prior assumptions on the effect size distributions of causal variants, their corresponding multivariate models (Cheng, Kizilkaya, *et al.* 2018) suffer from a high computational burden. In this paper, we developed a Bayesian sparse factor model with mixture priors on marker effects to implement both genome-wide prediction and association for analyses with hundreds to tens-of-thousands of phenotypes. We use a moderate number of latent factors (K) to account for the covariance among the observed traits. This substantially reduces the computational burden relative to either a multivariate Bayesian regression model or a MvLMM with fully parameterized trait covariance matrices when the number of traits (t) is large.

However, the sparse factor structure of MegaBayesC does not reduce the model complexity enough to enable mixture priors over the millions of genetic markers that are available in many systems from high-density genotyping arrays or whole genome sequencing. When marker effects of the factors and the trait-specific residuals are both included in the model, the number of marker effects to be estimated is equivalent to (t+K)×p, with t being the number of observed traits, K being the number of factors, and p being the number of total SNPs, which would require a tremendous amount of computational time and memory storage for whole-genome analysis.

We therefore developed 2 approximations to greatly reduce the time complexity of the full model. First, we forced the marker effects to affect the secondary traits through the K factors (although we do allow marker effects to independently control the focal trait). This reduces the number of marker effects to (K+1)p. Second, we developed a 2-stage approach to prune the candidate markers before subjecting the pruned markers to the MegaBayesC analysis. For our MegaBayesC analysis of the Arabidopsis dataset with n=649, t=20,844, and p=2,804, it took around 3 h to sample a MCMC chain of 10,000 iterations on a computer with 1 node and 20 CPU. Theoretically, the computational cost of MegaBayesC without fitting trait-specific marker effects (as in the analyses conducted in this paper) is approximately $O(K^{3}+K^{2}n+Knt+p^{3}+p^{2}n+pnK)$ if n>p, or $O(K^{3}+K^{2}n+Knt+n^{3}+pn^{2}+pnK)$ if p>n. Since K is generally much smaller than n, p, or t, the increase in complexity as a function of K is dominated by K(nt+pn) and so is approximately linear. As a function of t, the complexity is also linear. As a function of n and/or p, it is approximately quadratic-cubic in the smaller and linear in the larger because either a n×n or p×p matrix inversion is required.

While MegaBayesC, and MegaLMM more generally, shows promise in its ability to integrate thousands of traits in genome-wide prediction and association, the tradeoff between the benefit of incorporating secondary traits and the computational cost brought from the increased model complexity must be considered. Based on our simulated study, MegaBayesC can effectively disentangle the genetic and nongenetic sources of covariation among observed traits. When there is an important environmental component in the variation of focal trait, and this environmental component is shared by many other highly correlated traits, we expect MegaLMM whole-genome regression models to provide a large benefit by providing a tool to effectively control for this environmental variation. However, when the secondary traits are not highly correlated with the focal trait, or the heritability of the focal trait is already sufficiently high, MegaLMM whole-genome regression may prove less useful. In this paper, we have focused on 2 versions of MegaLMM: whole-genome regression with the BayesC prior on the marker effects (MegaBayesC), and with a ridge prior on the marker effects (MegaRRBLUP). The extension to other similar Bayesian Alphabet priors such as BayesA and BayesB would require only minor modifications to the code in the MegaLMM package. Other mixture priors such as the BayesR priors could also be implemented in future studies and we anticipate that each version may provide benefits in specific datasets.

## Supplementary Material

iyac183_Supplementary_Data

## Data Availability

Scripts for running all analyses are archived at GitHub: https://github.com/Jiayi-Qu/Mega-BayesC. The Bayesian Alphabet implementation is in the MegaLMM R package on GitHub. A stable release of the software used in this manuscript is available from Zenodo: https://doi.org/10.5281/zenodo.7158887. Data from the wheat breeding trial were downloaded from CIMMYT Research Data http://hdl.handle.net/11529/10548109 (Krause *et al.* 2019). Arabidopsis flowering time data were downloaded from Arapheno: https://arapheno.1001genomes.org/phenotype/261/. Gene expression data were downloaded from NCBI GEO: http://www.ncbi.nlm.nih.gov/geo/query/acc.cgi?acc=GSE80744 (Barrett *et al.* 2012). Genotype data were downloaded from the 1,001 genomes project (Alonso-Blanco *et al.* 2016). Supplemental material available at *GENETICS* online.
