# Supplementary material for: Mega-scale Bayesian regression methods for genome-wide prediction and association studies with thousands of traits
Source: Genetics. 2022 Dec 19;223(3):iyac183. doi: 10.1093/genetics/iyac183 (PMC9991502; doi:10.1093/genetics/iyac183)
Supplement: iyac183_Supplementary_Data [file iyac183_supplementary_data.pdf]

Mega-scale Bayesian Regression methods for genome-wide prediction and association studies with thousands of traits: Supplemental Document

1. GIBBS SAMPLER UPDATES

Sample F given all other parameters

To sample $\mathbf{F}$, we transpose Eq. 1:

$$\mathbf{Y}^T = \mathbf{\Lambda}^T\mathbf{F}^T + \mathbf{M}_R^T + \mathbf{E}_R^T \tag{S1}$$

where $\mathbf{M}_R = \mathbf{X}_1\mathbf{B}_1 + \mathbf{X}_{2R}\mathbf{B}_{2R}$. Conditioning on $\mathbf{B}_{2F}, \mathbf{B}_{2R}$, columns of $\mathbf{F}^T$ and $\mathbf{M}_R^T$ are uncorrelated and we can represent Eq. S1 as a set of simple linear regressions:

$$(\widetilde{\mathbf{Y}}^T)_i = \widetilde{\mathbf{\Lambda}}^T(\mathbf{F}^T)_i + (\widetilde{\mathbf{M}}_R^T)_i + (\widetilde{\mathbf{E}}_R^T)_i \tag{S2}$$

$$(\mathbf{F}^T)_i \sim N(\boldsymbol{\mu}_{(\mathbf{F}^T)_i}, \mathbf{D}_f) \tag{S3}$$

$$(\widetilde{\mathbf{E}}_R^T)_i \sim N(\mathbf{0}, \mathbf{D}_{(\widetilde{\mathbf{Y}}^T)_i}) \tag{S4}$$

where $\widetilde{\cdot}$ denotes the removal of missing trait data from the corresponding entity. For example, $(\widetilde{\mathbf{Y}}^T)_i$ is the sub-vector of non-missing traits in the ith row of $\mathbf{Y}$. $(\mathbf{F}^T)_i$ denotes the ith row of $\mathbf{F}$, which follows a multivariate normal distribution with mean $\boldsymbol{\mu}_{(\mathbf{F}^T)_i} = \mathbf{B}_{2F}^T(\mathbf{X}_{2F}^T)_i$ and (co)variance matrix $\mathbf{D}_f = \mathbf{\Psi}_{FE}$. $\mathbf{D}_{(\widetilde{\mathbf{Y}}^T)_i} = \widetilde{\mathbf{\Psi}}_{RE}$. $\mathbf{\Psi}_{FE}$ and $\widetilde{\mathbf{\Psi}}_{RE}$ are diagonal matrices.

Let $(\widetilde{\mathbf{Y}}_{cor}^T)_i = (\widetilde{\mathbf{Y}}^T)_i - (\widetilde{\mathbf{M}}_R^T)_i$, we have

$$(\widetilde{\mathbf{Y}}_{cor}^T)_i = \widetilde{\mathbf{\Lambda}}^T(\mathbf{F}^T)_i + (\widetilde{\mathbf{E}}_R^T)_i \tag{S5}$$

For simplicity, let $(\widetilde{\mathbf{Y}}_{cor}^T)_i = \mathbf{y}_{cor_i}$, $\widetilde{\mathbf{\Lambda}}^T = \mathbf{\Lambda}^T$, $(\mathbf{F}^T)_i = \mathbf{f}_i$, $\boldsymbol{\mu}_{(\mathbf{F}^T)_i} = \boldsymbol{\mu}_{f_i}$ and $\mathbf{D}_{(\widetilde{\mathbf{Y}}^T)_i} = \mathbf{D}_Y$. The full conditional posterior distribution for $(\mathbf{F}^T)_i$ is derived as:

$$\begin{aligned}
&f(\mathbf{f}_i|ELSE) \propto f(\mathbf{y}_{cor_i}|\mathbf{\Lambda}^T, \mathbf{f}_i, \mathbf{D}_Y)f(\mathbf{f}_i|\boldsymbol{\mu}_{f_i}, \mathbf{D}_f) \\
&\propto \exp\{-\frac{1}{2}(\mathbf{y}_{cor_i} - \mathbf{\Lambda}^T\mathbf{f}_i)^T(\mathbf{D}_Y)^{-1}(\mathbf{y}_{cor_i} - \mathbf{\Lambda}^T\mathbf{f}_i)\} \\
&\times \exp\{-\frac{1}{2}(\mathbf{f}_i - \boldsymbol{\mu}_{f_i})^T(\mathbf{D}_f)^{-1}(\mathbf{f}_i - \boldsymbol{\mu}_{f_i})\} \\
&\propto \exp\{-\frac{1}{2}(\mathbf{f}_i^T(\mathbf{D}_f^{-1} + \mathbf{\Lambda}\mathbf{D}_Y^{-1}\mathbf{\Lambda}^T)\mathbf{f}_i - 2(\mathbf{y}_{cor_i}^T\mathbf{D}_Y^{-1}\mathbf{\Lambda}^T + \boldsymbol{\mu}_{f_i}^T\mathbf{D}_f^{-1})\mathbf{f}_i)\} \\
&\propto \exp\{-\frac{1}{2}(\mathbf{f}_i^T\mathbf{C}\mathbf{f}_i - 2\mathbf{r}^T\mathbf{f}_i)\} \\
&\propto N(\mathbf{C}^{-1}\mathbf{r}, \mathbf{C}^{-1})
\end{aligned}$$

Therefore, $(\mathbf{F}^T)_i|ELSE \sim N(\boldsymbol{\mu}, \mathbf{\Sigma})$ with

$$\mathbf{\Sigma} = \left[\mathbf{D}_f^{-1} + \widetilde{\mathbf{\Lambda}}\mathbf{D}_{(\widetilde{\mathbf{Y}}^T)_i}^{-1}\widetilde{\mathbf{\Lambda}}^T\right]^{-1} \tag{S6}$$

$$\boldsymbol{\mu} = \mathbf{\Sigma}\left[\widetilde{\mathbf{\Lambda}}\mathbf{D}_{(\widetilde{\mathbf{Y}}^T)_i}^{-1}(\widetilde{\mathbf{Y}}_{cor}^T)_i + \mathbf{D}_f^{-1}\boldsymbol{\mu}_{(\mathbf{F}^T)_i}\right] \tag{S7}$$

Sample parameters in $\mathbf{\Lambda}$

A. Full conditional posterior distribution of $\mathbf{\Lambda}$

The prior for $\boldsymbol{\lambda}_j$ is specified as follows:

$$\boldsymbol{\lambda}_{kj} = \begin{cases} N(0, \tau_k^{-1}\sigma_{R_j}^2) & \text{probability } (1-\pi_k) \\ 0 & \text{probability } (\pi_k) \end{cases} \tag{S8}$$

$$\sigma_{R_j}^2 \sim iG(a_\sigma, b_\sigma) \tag{S9}$$

$$\tau_k = \prod_{h=1}^{k} \delta_h \tag{S10}$$

$$\delta_1 = 1, \quad \delta_h \sim Ga(a_\delta, b_\delta) \quad h = 2...k \tag{S11}$$

This mixture prior for $\boldsymbol{\lambda}_j$ can be parameterized as: $\mathbf{D}_{\gamma_j}\boldsymbol{\beta}_{\lambda_j}$, where $\mathbf{D}_{\gamma_j} = Diag(\boldsymbol{\gamma}_{\lambda_j})$ with

$$\gamma_{\lambda_j(k)} = \begin{cases} 1 & \text{probability } (1-\pi_{\Lambda_k}) \\ 0 & \text{probability } (\pi_{\Lambda_k}) \end{cases} \tag{S12}$$

and $\boldsymbol{\beta}_{\lambda_j} \sim N(\mathbf{0}, \sigma_{R_j}^2\mathbf{D}_\lambda = \sigma_{R_j}^2 Diag(\tau_k^{-1}))$ for k = 1, 2, ... K.

Conditional on $\mathbf{F}$, Eq. 1 can be simplified into t independent univariate linear mixed models for the columns of $\mathbf{Y}$. For the jth column of $\mathbf{Y}$:

$$\mathbf{y}_j = \mathbf{X}_1\mathbf{b}_{1j} + \mathbf{F}\mathbf{D}_{\gamma_j}\boldsymbol{\beta}_{\lambda_j} + \mathbf{X}_{2R}\mathbf{b}_{2R_j} + \mathbf{e}_{R_j} \tag{S13}$$

where $\mathbf{e}_{R_j} \sim N(\mathbf{0}, \sigma_{R_j}^2\mathbf{I})$.

$$\begin{aligned}
f(\boldsymbol{\beta}_{\lambda_j}|ELSE) &\propto f(\mathbf{y}_j|\mathbf{b}_{1j}, \mathbf{F}, \mathbf{D}_{\gamma_j}, \boldsymbol{\beta}_{\lambda_j}, \mathbf{b}_{2R_j}, \sigma_{R_j}^2) f(\boldsymbol{\beta}_{\lambda_j}|\mathbf{D}_\lambda, \sigma_{R_j}^2) \\
&\propto \exp\{-\frac{1}{2\sigma_{R_j}^2}(\boldsymbol{\epsilon} - \mathbf{F}\mathbf{D}_{\gamma_j}\boldsymbol{\beta}_{\lambda_j})^T(\boldsymbol{\epsilon} - \mathbf{F}\mathbf{D}_{\gamma_j}\boldsymbol{\beta}_{\lambda_j})\} \times \exp\{-\frac{1}{2\sigma_{R_j}^2}\boldsymbol{\beta}_{\lambda_j}^T\mathbf{D}_\lambda^{-1}\boldsymbol{\beta}_{\lambda_j}\} \\
&\propto \exp\{-\frac{1}{2}[\boldsymbol{\beta}_{\lambda_j}^T(\frac{\mathbf{D}_{\gamma_j}^T\mathbf{F}^T\mathbf{F}\mathbf{D}_{\gamma_j}}{\sigma_{R_j}^2} + \frac{\mathbf{D}_\lambda^{-1}}{\sigma_{R_j}^2})\boldsymbol{\beta}_{\lambda_j} - 2\frac{\boldsymbol{\epsilon}^T\mathbf{F}\mathbf{D}_{\gamma_j}}{\sigma_{R_j}^2}\boldsymbol{\beta}_{\lambda_j}]\} \\
&\propto \exp\{-\frac{1}{2}(\boldsymbol{\beta}_{\lambda_j}^T\mathbf{C}\boldsymbol{\beta}_{\lambda_j} - 2\mathbf{r}^T\boldsymbol{\beta}_{\lambda_j})\} \\
&\propto N(\mathbf{C}^{-1}\mathbf{r}, \mathbf{C}^{-1})
\end{aligned}$$

where $\boldsymbol{\epsilon} = \mathbf{y}_j - \mathbf{X}_1\mathbf{b}_{1j} - \mathbf{X}_{2R}\mathbf{b}_{2R_j}$, $\mathbf{C} = \frac{\mathbf{D}_{\gamma_j}^T\mathbf{F}^T\mathbf{F}\mathbf{D}_{\gamma_j} + \mathbf{D}_\lambda^{-1}}{\sigma_{R_j}^2}$, and $\mathbf{r} = \frac{\mathbf{D}_{\gamma_j}^T\mathbf{F}^T\boldsymbol{\epsilon}}{\sigma_{R_j}^2}$.

Besides the full conditional posterior distribution for the multivariate $\boldsymbol{\beta}_{\lambda_j}$ as derived above, a univariate version for the elements in $\boldsymbol{\beta}_{\lambda_j}$ is also derived as follows to prepare for the derivation of $\gamma_{\lambda_{kj}}$.

$$\begin{aligned}
f(\beta_{\lambda_{kj}}|ELSE) &\propto f(\mathbf{y}_j|\mathbf{b}_{1j}, \mathbf{F}, \mathbf{D}_{\gamma_j}, \boldsymbol{\beta}_{\lambda_j}, \mathbf{b}_{2R_j}, \sigma_{R_j}^2) f(\beta_{\lambda_{kj}}|\sigma_{R_j}^2, \tau_k) \\
&\propto \exp\{-\frac{1}{2\sigma_{R_j}^2}(\boldsymbol{\epsilon} - \sum_{i=1}^{K}\mathbf{F}_{\cdot i}\gamma_{\lambda_{ij}}\beta_{\lambda_{ij}})^T(\boldsymbol{\epsilon} - \sum_{i=1}^{K}\mathbf{F}_{\cdot i}\gamma_{\lambda_{ij}}\beta_{\lambda_{ij}})\} \times \exp\{-\frac{\tau_k\beta_{\lambda_{kj}}^2}{2\sigma_{R_j}^2}\} \\
&\propto \exp\{-\frac{1}{2\sigma_{R_j}^2}(\boldsymbol{\epsilon}^* - \mathbf{F}_{\cdot k}\gamma_{\lambda_{kj}}\beta_{\lambda_{kj}})^T(\boldsymbol{\epsilon}^* - \mathbf{F}_{\cdot k}\gamma_{\lambda_{kj}}\beta_{\lambda_{kj}})\} \times \exp\{-\frac{\tau_k\beta_{\lambda_{kj}}^2}{2\sigma_{R_j}^2}\} \\
&\propto \exp\{-\frac{1}{2}[(\frac{\mathbf{F}_{\cdot k}^T\mathbf{F}_{\cdot k}\gamma_{\lambda_{kj}}}{\sigma_{R_j}^2} + \frac{\tau_k}{\sigma_{R_j}^2})\beta_{\lambda_{kj}}^2 - 2\frac{\boldsymbol{\epsilon}^{*T}\mathbf{F}_{\cdot k}\gamma_{\lambda_{kj}}}{\sigma_{Rj}^2}\beta_{\lambda_{kj}}]\} \\
&\propto \exp\{-\frac{1}{2}[A_{kj}\beta_{\lambda_{kj}}^2 - 2r\beta_{\lambda_{kj}}]\} \\
&\propto N(A_{kj}^{-1}r, A_{kj}^{-1})
\end{aligned}$$

where $\boldsymbol{\epsilon}^* = \mathbf{y}_j - \mathbf{X}_1\mathbf{b}_{1j} - \mathbf{X}_{2R}\mathbf{b}_{2R_j} - \sum_{i=1, i\neq k}^{K}\mathbf{F}_{\cdot i}\gamma_{\lambda_{ij}}\beta_{\lambda_{ij}}$, $A_{kj} = \frac{\mathbf{F}_{\cdot k}^T\mathbf{F}_{\cdot k}\gamma_{\lambda_{kj}} + \tau_k}{\sigma_{Rj}^2}$, and $r = \frac{\boldsymbol{\epsilon}^{*T}\mathbf{F}_{\cdot k}\gamma_{\lambda_{kj}}}{\sigma_{Rj}^2}$.

B. Full conditional posterior distribution of $\gamma_{\lambda_{kj}}$

From the model specification, $\boldsymbol{\gamma}$ variables can take either 0 or 1. Let $\boldsymbol{\theta}$ denote all other parameters except for $\beta_{\lambda_{kj}}$ and $\gamma_{\lambda_{kj}}$, the marginal full conditional distribution of $\gamma_{\lambda_{kj}}$ that integrates $\beta_{\lambda_{kj}}$ is shown as:

$$f(\gamma_{\lambda_{kj}}|\boldsymbol{\theta},\mathbf{y}) = \frac{f(\gamma_{\lambda_{kj}},\boldsymbol{\theta},\mathbf{y})}{\sum_{\gamma_{\lambda_{kj}}} f(\gamma_{\lambda_{kj}},\boldsymbol{\theta},\mathbf{y})} \tag{S14}$$

$$= \frac{f(\mathbf{y}|\boldsymbol{\theta},\gamma_{\lambda_{kj}})f(\boldsymbol{\theta})f(\gamma_{\lambda_{kj}}|\pi_{\Lambda_k})}{\sum_{\gamma_{\lambda_{kj}}} f(\mathbf{y}|\boldsymbol{\theta},\gamma_{\lambda_{kj}})f(\boldsymbol{\theta})f(\gamma_{\lambda_{kj}}|\pi_{\Lambda_k})} \tag{S15}$$

$$= \frac{f(\mathbf{y}|\boldsymbol{\theta},\gamma_{\lambda_{kj}})f(\gamma_{\lambda_{kj}}|\pi_{\Lambda_k})}{\sum_{\gamma_{\lambda_{kj}}} f(\mathbf{y}|\boldsymbol{\theta},\gamma_{\lambda_{kj}})f(\gamma_{\lambda_{kj}}|\pi_{\Lambda_k})} \tag{S16}$$

Since $f(\mathbf{y}|\boldsymbol{\theta},\gamma_{\lambda_{kj}}) = \int f(\mathbf{y},\beta_{\lambda_{kj}}|\boldsymbol{\theta},\gamma_{\lambda_{kj}})d\beta_{\lambda_{kj}}$, the derivation for $f(\mathbf{y}|\boldsymbol{\theta},\gamma_{\lambda_{kj}})$ is shown as follows.

$$\begin{aligned}
&f(\mathbf{y}|\boldsymbol{\theta},\gamma_{\lambda_{kj}}) = \int f(\mathbf{y},\beta_{\lambda_{kj}}|\boldsymbol{\theta},\gamma_{\lambda_{kj}})d\beta_{\lambda_{kj}} \\
&= \int f(\mathbf{y}|\beta_{\lambda_{kj}},\boldsymbol{\theta},\gamma_{\lambda_{kj}})f(\beta_{\lambda_{kj}}|\sigma^2_{R_j},\tau_k)d\beta_{\lambda_{kj}} \\
&\propto \int \exp\{-\frac{1}{2}[(\frac{\mathbf{F}^T_{\cdot k}\mathbf{F}_{\cdot k}\gamma_{\lambda_{kj}}+\tau_k}{\sigma^2_{R_j}})\beta^2_{\lambda_{kj}} - 2\frac{\boldsymbol{\epsilon}^{*T}\mathbf{F}_{\cdot k}\gamma_{\lambda_{kj}}}{\sigma^2_{R_j}}\beta_{\lambda_{kj}}]\}d\beta_{\lambda_{kj}} \\
&\times \exp\{-\frac{\boldsymbol{\epsilon}^{*T}\boldsymbol{\epsilon}^*}{2\sigma^2_{R_j}}\} \\
&\propto \int \exp\{-\frac{1}{2}[A_{kj}\beta^2_{\lambda_{kj}} - 2r\beta_{\lambda_{kj}} + r^2A^{-1}_{kj}]\}d\beta_{\lambda_{kj}} \\
&\times \exp\{-\frac{1}{2}(\frac{\boldsymbol{\epsilon}^{*T}\boldsymbol{\epsilon}^*}{\sigma^2_{R_j}} - r^2A^{-1}_{kj})\} \\
&\propto \exp\{-\frac{1}{2}(\frac{\boldsymbol{\epsilon}^{*T}\boldsymbol{\epsilon}^*}{\sigma^2_{R_j}} - r^2A^{-1}_{kj})\}
\end{aligned}$$

where $\boldsymbol{\epsilon}^* = \mathbf{y}_j - \mathbf{X}_1\mathbf{b}_{1j} - \mathbf{X}_{2B}\mathbf{b}_{2R_j} - \sum_{i=1,i\neq k}^{K}\mathbf{F}_{\cdot i}\gamma_{\lambda_{ij}}\beta_{\lambda_{ij}}$, $A_{kj} = \frac{\mathbf{F}^T_{\cdot k}\mathbf{F}_{\cdot k}\gamma_{\lambda_{kj}}+\tau_k}{\sigma^2_{Rj}}$, and $r = \frac{\boldsymbol{\epsilon}^{*T}\mathbf{F}_{\cdot k}\gamma_{\lambda_{kj}}}{\sigma^2_{Rj}}$.

Given Eq. S16, we have

$$\begin{aligned}
f(\gamma_{\lambda_{kj}}=0) &= \frac{f(\mathbf{y}|\boldsymbol{\theta},\gamma_{\lambda_{kj}}=0)f(\gamma_{\lambda_{kj}}=0|\pi_{\Lambda_k})}{\sum_{\gamma_{\lambda_{kj}}} f(\mathbf{y}|\boldsymbol{\theta},\gamma_{\lambda_{kj}})f(\gamma_{\lambda_{kj}}|\pi_{\Lambda_k})} \\
&= \frac{\pi_{\Lambda_k}\times\exp\{\frac{1}{2}r^2A^{-1}_{kj}\}}{\sum_{\gamma_{\lambda_{kj}}}\pi_{\Lambda_k}\times\exp\{\frac{1}{2}r^2A^{-1}_{kj}\}} \\
&= \frac{\pi_{\Lambda_k}}{\pi_{\Lambda_k} + (1-\pi_{\Lambda_k})\exp\{\frac{1}{2}(\frac{\boldsymbol{\epsilon}^{*T}\mathbf{F}_{\cdot k}}{\sigma^2_{R_j}})^2(\frac{\mathbf{F}^T_{\cdot k}\mathbf{F}_{\cdot k}+\tau_k}{\sigma^2_{R_j}})^{-1}\}}
\end{aligned}$$

C. Full conditional posterior distribution of δ_l

In order to sample τ_k, we need to firstly sample δ_l when $K > 1$. To derive the full conditional posterior distribution of δ_l, vectorize $\mathbf{\Lambda}$ as $\boldsymbol{\lambda}$. Then, we have

$$\boldsymbol{\lambda} = \begin{bmatrix} \boldsymbol{\lambda}_1 \\ \cdots \\ \boldsymbol{\lambda}_t \end{bmatrix}_{Kt\times 1} \sim N(\mathbf{0}, \mathbf{\Sigma} =$$

$$\begin{bmatrix} \tau_1^{-1}\sigma^2_{R_1} & & & & & \\ & \ddots & & & & \\ & & \tau_K^{-1}\sigma^2_{R_1} & & & \\ & & & \tau_1^{-1}\sigma^2_{R_t} & & \\ & & & & \ddots & \\ & & & & & \tau_K^{-1}\sigma^2_{R_t} \end{bmatrix}_{Kt\times Kt})$$

Note that the determinant of a diagonal matrix is the product of elements of its diagonal.

$$\begin{aligned}
& f(\delta_l|ELSE) \propto f(\boldsymbol{\lambda}|\mathbf{\Sigma})f(\delta_l|a_\delta, b_\delta) \\
& \propto \prod_{k=1}^{K}\prod_{j=1}^{t}[(\tau_k)^{-1\times(-1/2)}\exp\{-\frac{1}{2}\frac{\lambda_{kj}^2}{\tau_k^{-1}\sigma^2_{R_j}}\}] \\
& \times (\delta_l)^{a_\delta - 1}\exp\{-b_\delta\delta_l\} \\
& \propto [\prod_{k=l}^{K}\prod_{j=1}^{t}(\delta_l)^{1/2}] \times \delta_l^{a_\delta-1} \times \exp\{-\frac{1}{2}\sum_{k=l}^{K}\sum_{j=1}^{t}\frac{\lambda_{kj}^2\tau_k}{\sigma^2_{R_j}}\}\exp\{-b_\delta\delta_l\} \\
& \propto [\prod_{k=l}^{K}\prod_{j=1}^{t}(\delta_l)^{1/2}] \times \delta_l^{a_\delta-1} \times \exp\{-b_\delta\delta_l\} \\
& \times \exp\{-\frac{1}{2}\sum_{k=l}^{K}\sum_{j=1}^{t}\frac{\lambda_{kj}^2(\prod_{h=1,h\neq l}^{k}\delta_h)\delta_l}{\sigma^2_{R_j}}\} \\
& \propto (\delta_l)^{\frac{t(K-l+1)}{2}+a_\delta-1}\exp\{-b_\delta\delta_l\} \\
& \times \exp\{-\frac{1}{2}[\sum_{k=l}^{K}(\prod_{h=1,h\neq l}^{k}\delta_h)\sum_{j=1}^{t}\frac{\lambda_{kj}^2}{\sigma^2_{R_j}}]\delta_l\} \\
& \propto Ga(a_\delta + \frac{t(K-l+1)}{2}, b_\delta + \frac{1}{2}\sum_{k=l}^{K}(\prod_{h=1,h\neq l}^{k}\delta_h)\sum_{j=1}^{t}\frac{\lambda_{kj}^2}{\sigma^2_{R_j}})
\end{aligned}$$

Parallel Model Setting

Given $\mathbf{F}$ and $\mathbf{\Lambda}$, although the design matrices may differ for columns of $\mathbf{Y}$ and $\mathbf{F}$, the form of both sets of conditional model can be similarly expressed as:

$$\mathbf{y} = \mathbf{X}_1\boldsymbol{\alpha} + \mathbf{X}_2\mathbf{D}_\gamma\boldsymbol{\beta} + \mathbf{e} \tag{S17}$$

where

$$\boldsymbol{\alpha} \sim N(\mathbf{0}, \infty) \tag{S18}$$

$$\boldsymbol{\beta} \sim N(0, \sigma_\beta^2 \mathbf{I}) \tag{S19}$$

$$\mathbf{D}_\gamma = Diag(\boldsymbol{\gamma}) \tag{S20}$$

$$\gamma_i = \begin{Bmatrix} 1 & \text{probability } (1-\pi) \\ 0 & \text{probability } (\pi) \end{Bmatrix} \tag{S21}$$

$$\mathbf{e} \sim N(\mathbf{0}, \sigma^2 \mathbf{I}) \tag{S22}$$

$$\sigma^2 \sim iG(a_0, b_0) \tag{S23}$$

$$\sigma_\beta^2 \sim iG(a_\beta, b_\beta) \tag{S24}$$

Conditional on $\mathbf{F}$ and $\boldsymbol{\Lambda}$, Eq. 1 can be simplified into t independent univariate linear mixed models for the columns of $\mathbf{Y}_{cor} = \mathbf{Y} - \mathbf{F}\boldsymbol{\Lambda}$:

$$\mathbf{y}_{cor_j} = \mathbf{X}_1 \mathbf{b}_{1_j} + \mathbf{X}_{2B} \boldsymbol{\beta}_{B2R_j} \circ \boldsymbol{\gamma}_{B2R_j} + \mathbf{e}_{R_j} \tag{S25}$$

where

$$\mathbf{b}_{1_j} \sim N(\mathbf{0}, \infty \mathbf{I}) \tag{S26}$$

$$\boldsymbol{\beta}_{B2R_j} \sim N(\mathbf{0}, \sigma_{B2R_j}^2 \mathbf{I}) \tag{S27}$$

$$\gamma_{B2R_{j(i)}} = \begin{cases} 1 & \text{probability } (1-\pi_j) \\ 0 & \text{probability } (\pi_j) \end{cases} \tag{S28}$$

$$\mathbf{e}_{R_j} \sim N(\mathbf{0}, \sigma_{R_j}^2 \mathbf{I}_n) \tag{S29}$$

Besides the columns of $\mathbf{Y}$, the columns of $\mathbf{F}$ can be similarly expressed into K independent univariate linear mixed models:

$$\mathbf{f}_k = \mathbf{X}_{2F} \boldsymbol{\beta}_{B2F_k} \circ \boldsymbol{\gamma}_{B2F_k} + \mathbf{e}_{F_k} \tag{S30}$$

where

$$\boldsymbol{\beta}_{B2F_k} \sim N(\mathbf{0}, \sigma_{B2F_k}^2 \mathbf{I}) \tag{S31}$$

$$\gamma_{B2F_{k(i)}} = \begin{cases} 1 & \text{probability } (1-\pi_{F_k}) \\ 0 & \text{probability } (\pi_{F_k}) \end{cases} \tag{S32}$$

$$\mathbf{e}_{F_k} \sim N(\mathbf{0}, \sigma_{F_k}^2 \mathbf{I}_n) \tag{S33}$$

$$\tag{S34}$$

Here, factor-specific and trait-specific prior on the marker exclusion probability (π_{F_k} and π_j) and the variance of marker effects ($\sigma_{B2F_k}^2$ and $\sigma_{B2R_j}^2$) are used for each latent factor and observed trait. We can see that the columns of $\mathbf{Y}$ and $\mathbf{F}$ can be generally expressed by Eq. S17. That is, for columns of $\mathbf{Y}$, $\mathbf{y} = \mathbf{y}_{cor_j}$, $\boldsymbol{\alpha} = \mathbf{b}_{1_j}$, $\mathbf{D}_\gamma = Diag(\boldsymbol{\gamma}_{B2R_j})$, $\boldsymbol{\beta} = \boldsymbol{\beta}_{B2R_j}$, $\mathbf{e} = \mathbf{e}_{R_j}$, $\sigma^2 = \sigma_{R_j}^2$, $\sigma_\beta^2 = \sigma_{B2R_j}^2$. Similarly, for columns of $\mathbf{F}$, $\mathbf{y} = \mathbf{f}_k$, $\boldsymbol{\alpha}$ is empty, $\mathbf{D}_\gamma = Diag(\boldsymbol{\gamma}_{B2F_k})$, $\boldsymbol{\beta} = \boldsymbol{\beta}_{B2F_k}$, $\mathbf{e} = \mathbf{e}_{F_k}$, $\sigma^2 = \sigma_{F_k}^2$, $\sigma_\beta^2 = \sigma_{B2F_k}^2$. Furthermore, we defined the following term based on the notation in Eq. S17:

$$\mathbf{V}_\beta = \mathbf{X}_2 \mathbf{D}_\gamma \mathbf{X}_2^T \sigma_\beta^2 + \sigma^2 \mathbf{I}$$

D. Full conditional posterior distribution of $\boldsymbol{\alpha}$

The conditional posterior distribution for $\boldsymbol{\alpha}$(*i.e.*, $\mathbf{b}_{1j}$) is derived as (integrating out $\boldsymbol{\beta}$):

$$
\begin{aligned}
f(\boldsymbol{\alpha}|\cdot) &\propto f(\mathbf{y}|\boldsymbol{\alpha},\mathbf{V}_\beta)\\
&\propto \exp\{-\frac{1}{2}(\mathbf{y}-\mathbf{X}_1\boldsymbol{\alpha})^T\mathbf{V}_\beta^{-1}(\mathbf{y}-\mathbf{X}_1\boldsymbol{\alpha})\}\\
&\propto \exp\{-\frac{1}{2}(\boldsymbol{\alpha}^T\mathbf{X}_1^T\mathbf{V}_\beta^{-1}\mathbf{X}_1\boldsymbol{\alpha}-2\mathbf{y}^T\mathbf{V}_\beta^{-1}\mathbf{X}_1\boldsymbol{\alpha})\}\\
&\propto \exp\{-\frac{1}{2}(\boldsymbol{\alpha}^T\mathbf{A}_\alpha\boldsymbol{\alpha}-2\mathbf{r}^T\boldsymbol{\alpha})\}\\
&\propto N(\mathbf{A}_\alpha^{-1}\mathbf{r},\mathbf{A}_\alpha^{-1})
\end{aligned}
$$

where $\mathbf{A}_\alpha = \mathbf{X}_1^T\mathbf{V}_\beta^{-1}\mathbf{X}_1$, $\mathbf{r} = \mathbf{X}_1^T\mathbf{V}_\beta^{-1}\mathbf{y}$. The dimension of $\mathbf{A}_\alpha$ is $a(b_1)\times a(b_1)$, and the dimension of $\mathbf{V}_\beta$ is $n \times n$.

E. Full conditional posterior distribution of σ^2

The conditional posterior distribution for $\sigma^2(i.e., \sigma^2_{R_j}$ and $\sigma^2_{F_k})$ is derived as:

$$
\begin{aligned}
f(\sigma^2|\cdot) &\propto f(\mathbf{y}|\boldsymbol{\alpha},\mathbf{D}_\gamma,\boldsymbol{\beta},\sigma^2)f(\sigma^2|a_0,b_0)\\
&\propto (\sigma^2)^{-\frac{n}{2}}\exp\{-\frac{1}{2\sigma^2}(\mathbf{y}-\mathbf{X}_1\boldsymbol{\alpha}-\mathbf{X}_2\mathbf{D}_\gamma\boldsymbol{\beta})^T(\mathbf{y}-\mathbf{X}_1\boldsymbol{\alpha}-\mathbf{X}_2\mathbf{D}_\gamma\boldsymbol{\beta})\}\\
&\times (\sigma^2)^{-a_0-1}\exp\{-\frac{b_0}{\sigma^2}\}\\
&\propto (\sigma^2)^{-(\frac{n}{2}+a_0)-1}\exp\{-\frac{\boldsymbol{\epsilon}^T\boldsymbol{\epsilon}/2}{\sigma^2}\}\exp\{-\frac{b_0}{\sigma^2}\}\\
&\propto iG(\frac{n}{2}+a_0,\frac{\boldsymbol{\epsilon}^T\boldsymbol{\epsilon}}{2}+b_0)
\end{aligned}
$$

where $\boldsymbol{\epsilon} = \mathbf{y}-\mathbf{X}_1\boldsymbol{\alpha}-\mathbf{X}_2\mathbf{D}_\gamma\boldsymbol{\beta}$.

F. Full conditional posterior distribution of $\boldsymbol{\beta}$

The conditional posterior distribution for $\boldsymbol{\beta}$ is derived as:

$$
\begin{aligned}
f(\boldsymbol{\beta}|\cdot) &\propto f(\mathbf{y}|\boldsymbol{\alpha},\mathbf{D}_\gamma,\boldsymbol{\beta},\sigma^2)f(\boldsymbol{\beta}|\sigma^2_\beta)\\
&\propto \exp\{-\frac{1}{2\sigma^2}(\mathbf{y}-\mathbf{X}_1\boldsymbol{\alpha}-\mathbf{X}_2\mathbf{D}_\gamma\boldsymbol{\beta})^T(\mathbf{y}-\mathbf{X}_1\boldsymbol{\alpha}-\mathbf{X}_2\mathbf{D}_\gamma\boldsymbol{\beta})\}\\
&\times \exp\{-\frac{1}{2\sigma^2_\beta}\boldsymbol{\beta}^T\boldsymbol{\beta}\}\\
&\propto \exp\{-\frac{1}{2\sigma^2}(\boldsymbol{\epsilon}-\mathbf{X}_2\mathbf{D}_\gamma\boldsymbol{\beta})^T(\boldsymbol{\epsilon}-\mathbf{X}_2\mathbf{D}_\gamma\boldsymbol{\beta})\}\times\exp\{-\frac{1}{2\sigma^2_\beta}\boldsymbol{\beta}^T\boldsymbol{\beta}\}\\
&\propto \exp\{-\frac{1}{2}(\boldsymbol{\beta}^T(\frac{\mathbf{D}_\gamma^T\mathbf{X}_2^T\mathbf{X}_2\mathbf{D}_\gamma}{\sigma^2}+\frac{1}{\sigma^2_\beta}\mathbf{I})\boldsymbol{\beta}-2\frac{\boldsymbol{\epsilon}^T\mathbf{X}_2\mathbf{D}_\gamma}{\sigma^2}\boldsymbol{\beta})\}\\
&\propto \exp\{-\frac{1}{2}(\boldsymbol{\beta}^T\mathbf{A}_\beta\boldsymbol{\beta}-2\mathbf{r}^T\boldsymbol{\beta})\}\\
&\propto N(\mathbf{A}_\beta^{-1}\mathbf{r},\mathbf{A}_\beta^{-1})
\end{aligned}
$$

where $\mathbf{A}_\beta = \frac{\mathbf{D}_\gamma^T\mathbf{X}_2^T\mathbf{X}_2\mathbf{D}_\gamma}{\sigma^2}+\frac{1}{\sigma^2_\beta}\mathbf{I}$, $\mathbf{r} = \frac{\mathbf{D}_\gamma^T\mathbf{X}_2^T\boldsymbol{\epsilon}}{\sigma^2}$. The dimension of $\mathbf{A}_\beta$ is $b\times b$. For columns of $\mathbf{Y}$, $b = b_{2R}$, $\boldsymbol{\epsilon} = \mathbf{y}_{cor_j}-\mathbf{X}_1\mathbf{b}_{1_j}$. For columns of $\mathbf{F}$, $b = b_{2F}$, $\boldsymbol{\epsilon} = \mathbf{f}_k$. Besides the full conditional posterior distribution of the multivariate $\boldsymbol{\beta}$ as derived above, a univariate version for the elements β_l in $\boldsymbol{\beta}$ is also written as follows.

$$
\begin{aligned}
&f(\beta_l|\cdot) \propto f(\mathbf{y}|\boldsymbol{\alpha}, \mathbf{D}_\gamma, \boldsymbol{\beta}, \sigma^2) f(\beta_l|\sigma_\beta^2) \\
&\propto \exp\{-\frac{1}{2\sigma^2}(\mathbf{y} - \mathbf{X}_1\boldsymbol{\alpha} - \sum_{i=1}^{b} \mathbf{X}_{2\cdot i}\gamma_i\beta_i)^T(\mathbf{y} - \mathbf{X}_1\boldsymbol{\alpha} - \sum_{i=1}^{b} \mathbf{X}_{2\cdot i}\gamma_i\beta_i)\} \\
&\times \exp\{-\frac{\beta_l^2}{2\sigma_\beta^2}\} \\
&\propto \exp\{-\frac{1}{2\sigma^2}(\boldsymbol{\epsilon} - \mathbf{X}_{2\cdot l}\gamma_l\beta_l)^T(\boldsymbol{\epsilon} - \mathbf{X}_{2\cdot l}\gamma_l\beta_l)\} \exp\{-\frac{\beta_l^2}{2\sigma_\beta^2}\} \\
&\propto \exp\{-\frac{1}{2}[(\frac{\mathbf{X}_{2\cdot l}^T\mathbf{X}_{2\cdot l}\gamma_l}{\sigma^2} + \frac{1}{\sigma_\beta^2})\beta_l^2 - 2\frac{\boldsymbol{\epsilon}^T\mathbf{X}_{2\cdot l}\gamma_l}{\sigma^2}\beta_l]\} \\
&\propto exp\{-\frac{1}{2}(A_\beta\beta_l^2 - 2r\beta_l)\} \\
&\propto N(A_\beta^{-1}r, A_\beta^{-1})
\end{aligned}
$$

where $\boldsymbol{\epsilon} = \mathbf{y} - \mathbf{X}_1\boldsymbol{\alpha} - \sum_{i=1,i\neq l}^{b} \mathbf{X}_{2\cdot i}\gamma_i\beta_i$, $A_\beta = \frac{\mathbf{X}_{2\cdot l}^T\mathbf{X}_{2\cdot l}\gamma_l}{\sigma^2} + \frac{1}{\sigma_\beta^2}$, $r = \frac{\boldsymbol{\epsilon}^T\mathbf{X}_{2\cdot l}\gamma_l}{\sigma^2}$.

G. Full conditional posterior distribution of γ_l

Let $\boldsymbol{\theta}$ denote all other parameters except for β_l and γ_l, the marginal full conditional distribution of γ_l that integrates out β_l is shown as:

$$
f(\gamma_l|\boldsymbol{\theta}, \mathbf{y}) = \frac{f(\gamma_l, \boldsymbol{\theta}, \mathbf{y})}{\sum_{\gamma_l} f(\gamma_l, \boldsymbol{\theta}, \mathbf{y})} \tag{S35}
$$

$$
= \frac{f(\mathbf{y}|\boldsymbol{\theta}, \gamma_l) f(\boldsymbol{\theta}) f(\gamma_l|\pi)}{\sum_{\gamma_l} f(\mathbf{y}|\boldsymbol{\theta}, \gamma_l) f(\boldsymbol{\theta}) f(\gamma_l|\pi)} \tag{S36}
$$

$$
= \frac{f(\mathbf{y}|\boldsymbol{\theta}, \gamma_l) f(\gamma_l|\pi)}{\sum_{\gamma_l} f(\mathbf{y}|\boldsymbol{\theta}, \gamma_l) f(\gamma_l|\pi)} \tag{S37}
$$

Since $f(\mathbf{y}|\boldsymbol{\theta}, \gamma_l) = \int f(\mathbf{y}, \beta_l|\boldsymbol{\theta}, \gamma_l)d\beta_l$, the derivation for $f(\mathbf{y}|\boldsymbol{\theta}, \gamma_l)$ is shown as follows.

$$
\begin{aligned}
&f(\mathbf{y}|\boldsymbol{\theta}, \gamma_l) = \int f(\mathbf{y}, \beta_l|\boldsymbol{\theta}, \gamma_l)d\beta_l \\
&= \int f(\mathbf{y}|\beta_l, \boldsymbol{\theta}, \gamma_l) f(\beta_l|\sigma_\beta^2)d\beta_l \\
&\propto \int \exp\{-\frac{1}{2\sigma^2}(\boldsymbol{\epsilon} - \mathbf{X}_{2\cdot l}\gamma_l\beta_l)^T(\boldsymbol{\epsilon} - \mathbf{X}_{2\cdot l}\gamma_l\beta_l)\} \exp\{-\frac{\beta_l^2}{2\sigma_\beta^2}\}d\beta_l \\
&\propto \int (A_\beta)^{-\frac{1}{2}} \exp\{-\frac{1}{2}[(\frac{\mathbf{X}_{2\cdot l}^T\mathbf{X}_{2\cdot l}\gamma_l}{\sigma^2} + \frac{1}{\sigma_\beta^2})\beta_l^2 - 2\frac{\boldsymbol{\epsilon}^T\mathbf{X}_{2\cdot l}\gamma_l}{\sigma^2}\beta_l]\}d\beta_l \times \exp\{-\frac{1}{2\sigma^2}\boldsymbol{\epsilon}^T\boldsymbol{\epsilon}\} \\
&\propto \int (A_\beta)^{-\frac{1}{2}} \exp\{-\frac{1}{2}(A_\beta\beta_l^2 - 2r\beta_l + r^2A_\beta^{-1})\}d\beta_l \times (A_\beta)^{-\frac{1}{2}} \exp\{\frac{1}{2}r^2A_\beta^{-1}\} \times \exp\{-\frac{1}{2\sigma^2}\boldsymbol{\epsilon}^T\boldsymbol{\epsilon}\} \\
&\propto (A_\beta)^{-\frac{1}{2}} \exp\{\frac{1}{2}r^2A_\beta^{-1}\} \times \exp\{-\frac{1}{2\sigma^2}\boldsymbol{\epsilon}^T\boldsymbol{\epsilon}\}
\end{aligned}
$$

where $\boldsymbol{\epsilon} = \mathbf{y} - \mathbf{X}_1\boldsymbol{\alpha} - \sum_{i=1,i\neq l}^{b} \mathbf{X}_{2\cdot i}\gamma_i\beta_i$, $A_\beta = \frac{\mathbf{X}_{2\cdot l}^T\mathbf{X}_{2\cdot l}\gamma_l}{\sigma^2} + \frac{1}{\sigma_\beta^2}$, $r = \frac{\boldsymbol{\epsilon}^T\mathbf{X}_{2\cdot l}\gamma_l}{\sigma^2}$.

Given Eq. S37, we have

$$
\begin{aligned}
f(\gamma_l = 0) &= \frac{\pi \times (A_\beta)^{-\frac{1}{2}} \times \exp\{\frac{1}{2}r^2A_\beta^{-1}\}}{\sum_{\gamma_l} \pi_{\gamma_l} \times (A_\beta)^{-\frac{1}{2}} \times \exp\{\frac{1}{2}r^2A_\beta^{-1}\}} \\
&= \frac{\pi \times \sigma_\beta}{\pi \times \sigma_\beta + (1-\pi) \times (\frac{\mathbf{X}_{2\cdot l}^T\mathbf{X}_{2\cdot l}}{\sigma^2} + \frac{1}{\sigma_\beta^2})^{-\frac{1}{2}} \times \exp\{\frac{1}{2}(\frac{\boldsymbol{\epsilon}^T\mathbf{X}_{2\cdot l}}{\sigma^2})^2(\frac{\mathbf{X}_{2\cdot l}^T\mathbf{X}_{2\cdot l}}{\sigma^2} + \frac{1}{\sigma_\beta^2})^{-1}\}}
\end{aligned}
$$

$$f(\gamma_l = 1) = \frac{(1-\pi) \times (\frac{\mathbf{X}_{2\cdot l}^T \mathbf{X}_{2\cdot l}}{\sigma^2} + \frac{1}{\sigma_\beta^2})^{-\frac{1}{2}} \times \exp\{\frac{1}{2}(\frac{\boldsymbol{\epsilon}^T \mathbf{X}_{2\cdot l}}{\sigma^2})^2 (\frac{\mathbf{X}_{2\cdot l}^T \mathbf{X}_{2\cdot l}}{\sigma^2} + \frac{1}{\sigma_\beta^2})^{-1}\}}{\pi \times \sigma_\beta + (1-\pi) \times (\frac{\mathbf{X}_{2\cdot l}^T \mathbf{X}_{2\cdot l}}{\sigma^2} + \frac{1}{\sigma_\beta^2})^{-\frac{1}{2}} \times \exp\{\frac{1}{2}(\frac{\boldsymbol{\epsilon}^T \mathbf{X}_{2\cdot l}}{\sigma^2})^2 (\frac{\mathbf{X}_{2\cdot l}^T \mathbf{X}_{2\cdot l}}{\sigma^2} + \frac{1}{\sigma_\beta^2})^{-1}\}}$$

H. Full conditional posterior distribution of σ_β^2

$$\begin{aligned}
&f(\sigma_\beta^2) \propto f(\boldsymbol{\beta}|\sigma_\beta^2) f(\sigma_\beta^2|a_\beta, b_\beta) \\
&\propto (\sigma_\beta^2)^{-\frac{b}{2}} \exp\{-\frac{1}{2\sigma_\beta^2}\boldsymbol{\beta}^T\boldsymbol{\beta}\} \times (\sigma_\beta^2)^{-a_\beta - 1} \exp\{-\frac{b_\beta}{\sigma_\beta^2}\} \\
&\propto (\sigma_\beta^2)^{-\frac{b}{2} - a_\beta - 1} \exp\{-\frac{1}{\sigma_\beta^2}(\frac{\boldsymbol{\beta}^T\boldsymbol{\beta}}{2} + b_\beta)\} \\
&\propto iG(\frac{b}{2} + a_\beta, \frac{\boldsymbol{\beta}^T\boldsymbol{\beta}}{2} + b_\beta)
\end{aligned}$$

2. SPECIFICATION OF PARAMETERS FOR THE REAL DATA ANALYSIS PERFORMED IN THE PAPER

Model (Analysis)	K	Chain Length	Burn-in
MegaBayesC (GP)	100	10K	2K
MegaGBLUP (GP)	100	10K	2K
MegaRRBLUP (GP)	100	10K	2K
MegaBayesC (GWAS)	100	80K	20K

3. HYPERPARAMETERS FOR PRIOR DISTRIBUTIONS USED IN MEGABAYESC OR MEGARRBLUP

The elicitation of priors used in MegaBayesC and MegaRRBLUP is modified from that proposed in [1]. The priors used in MegaGBLUP stay the same as in [1].

Parameter	Distribution	Hyperparameters
$\sigma^2_{R_j}$	iG($a_\sigma = \nu - 1, b_\sigma = 1/(V\nu)$)	$\nu = 10, V = 0.5$
$\sigma^2_{F_k}$	iG($a_\sigma = \nu - 1, b_\sigma = 1/(V\nu)$)	$\nu = 10000, V = 0.9$
δ_h	Gamma(a_δ, b_δ)	$a_\delta = 1, b_\delta = 3$
$\sigma^2_{b2F_k}$	iG($\alpha = \nu/2, \beta = \nu\tau^2/2$)	$\nu = 4, \tau^2 = 1$

A inverse-gamma prior distribution is used for $\sigma^2_{R_j}/\sigma^2_{F_k}$, whose mean is equivalent to $\frac{1}{V}$, which indicates the proportion of variance explained by trait/factor residuals. In addition, a parameter ν is used to regulate the mixing property of these prior distributions. A gamma distribution is used to $delta_h$ with its mean equivalent to $a_\delta b_\delta = 3$. Through this prior specification, the magnitude of δ_h stochastically increases from one latent factor to the next, and thus, variance of factor loadings stochastically decreases as factor index increases. A scaled inverse chi-squared distribution is used for $\sigma^2_{b2F_k}$ with scale parameters = 1 and degree of freedom = 4.

4. SUPPLEMENTARY PLOTS

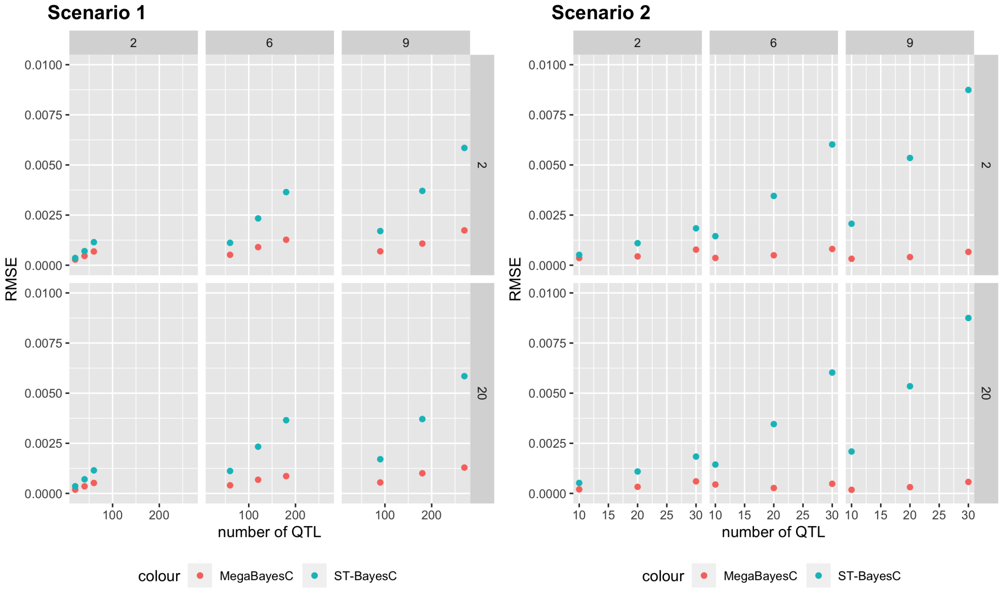

Fig. S1. RMSE of estimated marker effects under two scenarios for the total 36 different simulation settings. The performance of single-trait BayesC and MegaBayesC were compared. The performance of models for the simulation setting with n_{trait} = 2 and 20 are presented at the first and second row, respectively. The performance of models for the simulation setting with n_{factor} = 2, 6, 9 are presented at the first, second, and third column, respectively.

REFERENCES

1. D. E. Runcie, J. Qu, H. Cheng, and L. Crawford, “Megalmm: Mega-scale linear mixed models for genomic predictions with thousands of traits,” Genome Biol. **22**, 1–25 (2021).
